# An old model with new insights: endogenous retroviruses drive the evolvement toward ASD susceptibility and hijack transcription machinery during development

**DOI:** 10.1038/s41380-023-01999-z

**Published:** 2023-03-07

**Authors:** Chia-Wen Lin, Jacob Ellegood, Kota Tamada, Ikuo Miura, Mikiko Konda, Kozue Takeshita, Koji Atarashi, Jason P. Lerch, Shigeharu Wakana, Thomas J. McHugh, Toru Takumi

**Affiliations:** 1 Laboratory for Mental Biology, RIKEN Brain Science Institute, Wako, 351-0198 Saitama Japan; 2 Laboratory for Circuit and Behavioral Physiology, RIKEN Center for Brain Science, Wako, 351-0198 Saitama Japan; 3 Department of Physiology and Cell Biology, Kobe University School of Medicine, Chuo, 650-0017 Kobe Japan; 4 Mouse Imaging Centre, Hospital for Sick Children, Toronto, Ontario M5T 3H7 Canada; 5 Technology and Development Team for Mouse Phenotype Analysis, Japan Mouse Clinic, RIKEN BioResource Research Center, Tsukuba, Ibaraki 305-0074 Japan; 6 Department of Microbiology and Immunology, Keio University School of Medicine, Shinjuku, 160-8582 Tokyo Japan; 7 RIKEN Center for Integrative Medical Sciences, Tsurumi, 230-0045 Yokohama Japan; 8 Wellcome Centre for Integrative Neuroimaging, University of Oxford, Oxford, Oxfordshire OX39DU UK; 9 RIKEN Center for Biosystems Dynamics Research, Chuo, 650-0047 Kobe Japan

**Keywords:** Neuroscience, Autism spectrum disorders

## Abstract

The BTBR *T*^+^*Itpr3*^*tf*^/J (BTBR/J) strain is one of the most valid models of idiopathic autism, serving as a potent forward genetics tool to dissect the complexity of autism. We found that a sister strain with an intact corpus callosum, BTBR TF/ArtRbrc (BTBR/R), showed more prominent autism core symptoms but moderate ultrasonic communication/normal hippocampus-dependent memory, which may mimic autism in the high functioning spectrum. Intriguingly, disturbed epigenetic silencing mechanism leads to hyperactive endogenous retrovirus (ERV), a mobile genetic element of ancient retroviral infection, which increases de novo copy number variation (CNV) formation in the two BTBR strains. This feature makes the BTBR strain a still evolving multiple-loci model toward higher ASD susceptibility. Furthermore, active ERV, analogous to virus infection, evades the integrated stress response (ISR) of host defense and hijacks the transcriptional machinery during embryonic development in the BTBR strains. These results suggest dual roles of ERV in the pathogenesis of ASD, driving host genome evolution at a long-term scale and managing cellular pathways in response to viral infection, which has immediate effects on embryonic development. The wild-type Draxin expression in BTBR/R also makes this substrain a more precise model to investigate the core etiology of autism without the interference of impaired forebrain bundles as in BTBR/J.

## Introduction

Autism spectrum disorder (ASD) is a neurodevelopmental disorder with complex genetic architecture and heterogeneity, which have impeded the development of therapeutic strategies for this disease with a 2.3% prevalence rate in 2018 [1]. Studies based on monogenic ASD, either from rare or de novo mutation, have confirmed the category of synaptic dysfunction in the pathogenesis of ASD and have accumulated a growing list of ASD-risk genes [2]. However, these syndromic genes account only for 10–20% of all ASD cases, which suggests the potential entanglement of genetic susceptibility variants and epigenetic effects contributed by environmental factors [3]. Single nucleotide polymorphisms (SNPs) or single nucleotide variations (SNVs) are the most frequently observed genetic variation in the genome but have a more limited probability of affecting gene function. Instead, accumulating evidence suggests a prominent role of copy number variation (CNV), large-scale structural variations in the chromosome, contributing either directly to ASD pathology or ASD susceptibility [4, 5]. These observations suggest the niche of the forward genetic approach in accessing the core of major ASD cases.

From a phenotyping screen among several inbred strains, BTBR *T*^+^*Itpr3*^*tf*^/J (hereafter referred to as BTBR/J) has been recognized as an idiopathic model which well recapitulates the core symptoms of autism, including impairments in social interaction, repetitive behaviors, and often associated ultrasonic communication deficits [6–8]. It is interesting to note that inbred strains genetically close to BTBR/J, such as 129 × 1/SvJ strain [6, 8], show similar autism-like behavioral features, suggesting the existence of autism genetic susceptibility among mouse inbred strains. By using a sister strain of BTBR TF/ArtRbrc (hereafter referred to as BTBR/R), we recently discovered a disturbed epigenetic mechanism leads to the pathologic origins of systemic immune dysregulation in this strain by affecting definitive hematopoiesis in the yolk sac and aorta-gonad-mesonephros [9]. Together, these results suggest the BTBR strain as a potential model to investigate the multiple-hit theory of autism [2, 4]. BTBR/R and BTBR/J are derived from a common inbred strain BTBR and were deposited to RIKEN BioResource Research Center in 1987 and The Jackson Laboratory in 1994, respectively [10, 11]. However, substantial variations, including brain anatomy, behaviors, and immune phenotype, have accumulated between the two strains after ~30-year separation, a relatively short time in terms of strain evolution. The accelerated strain segregation suggests an unknown mechanism leading to genome instability between BTBR/R and BTBR/J. To identify the motivating force behind this, we compared the CNV composition between the two BTBR strains, which is the most “efficient” genetic variation to affect gene expression. Intriguingly, by analyzing the repeat sequences in the identified CNV, we found the potential involvement of endogenous retrovirus (ERV) in speeding up CNV formation in both BTBR strains. Furthermore, by single-cell RNA sequencing (scRNA seq), evidence of ERV activation during embryonic development was identified. These results suggest ongoing events of viral evasion centering on the host integrated stress response (ISR), which leads to a global alteration in the transcriptome of BTBR mice. These results unravel the idiopathic etiology of the BTBR strain by suggesting it as a superimposed model of autism genetic susceptibility and endogenous virus infection. The ancient viral infection and reactivation affect host genome instability in the long term and have a continuing effect on embryonic development. With the new advance in this old model, our study provides insights into how ASD susceptibility evolves in the genome and suggests BTBR/R as a precise model to investigate the core etiology of autism.

## Material and method

### Animals

C57BL/6J and two BTBR strains, BTBRTF/ArtRbrc (BTBR/R) (strain no, RBRC01206) and BTBR *T*^+^*Itpr3*^*tf*^/J (BTBR/J) (strain no, 002282), were purchased from Japan SLC Inc. (Hamamatsu, Japan), RIKEN BioResource Research Center (Tsukuba, Japan), and the Jackson Laboratory, respectively. All three strains were maintained in the same breeding room under controlled temperature at 23 ± 0.5 °C and humidity of 50–60% with 12 h light-dark cycle (light on 8 am) and ad libitum access to water and food. All procedures for animal handling followed the Animal Experimentation Committee of RIKEN Brain Science Institute guidelines.

### Array-based comparative genomic hybridization (aCGH)

aCGH was performed according to the manufacture’s protocol (SurePrint G3 Mouse CGH Microarray Kit, 1 × 1 M, #G4838A, Agilent Technologies). Briefly, genomic DNA (gDNA) was extracted from the tails of male mice by using Blood & Cell Culture DNA Mini Kit (Qiagen). The quality of DNA was checked for the absorbance at 320 nm, the ratio of OD260/280 > 1.8 and OD260/230 > 1.0. The gDNA was digested and labeled with Cy3- or Cy5-dCTP by random priming (BioPrime DNA Labeling Kit, Invitrogen). After hybridization, the fluorescence signals were scanned and analyzed by using DNAcopy, Gviz, and GenomicRanges packages under the R environment [12].

### Magnetic resonance imaging (MRI)

Nine-week-old male mice of both BTBR strains and B6 were used for the MRI study. The mice were anesthetized for transcardiac perfusion at the speed of 1 ml solution per minute. They were first flushed with 30 ml of PBS containing 1 μl/ml heparin, 2 mM ProHance, and then fixed by 30 ml of 4% paraformaldehyde in PBS with 2 mM ProHance at room temperature. The brain remained in the skull with the tissue, zygomatic bone, and the lower jaw removed. After dissection, the remaining skull structure was placed in a 15 ml tube filled with fixation solution overnight at 4 °C. On the next day, each specimen was preserved in PBS with 0.02% sodium azide and 2 mM ProHance and kept at 4 °C for at least one month until MRI scanning [13]. A multi-channel 7.0 Tesla MRI scanner (Agilent, Palo Alto, CA) was used to image the brains within their skulls. Sixteen custom-built solenoid coils were used to image the brains in parallel [14].

#### Anatomical scan

In order to detect volumetric changes, the following parameters were used for the MRI scan, which was a T2- weighted, 3-D fast spin-echo sequence with a cylindrical acquisition of k-space, TR of 350 ms, TEs of 12 ms per echo for six echoes, field-of-view equaled to 20 × 20 × 25 mm^3^ and matrix size equaled to 504 × 504 × 630. Voxel dimensions for this scan were 0.040 mm isotropic. The total imaging time was ~14 h.

#### Diffusion Tensor Imaging

Diffusion Tensor Imaging (DTI) was done using a 3D diffusion weighted fast spin echo sequence, with an echo train length of 6. Parameters for the DTI sequences were TR = 270 ms, first TE = 32 ms, and a TE of 10 ms for the remaining five echoes, 1 average. Field-of-view of 14 × 14 × 25 mm^3^ and a matrix size of 180 × 180 × 324 yielding an image with 78 μm isotropic voxels. Five *b* = 0 s/mm^2^ images and 30 high *b*-value (*b* = 2147 s/mm^2^) images in 30 different directions were acquired using the Jones30 scheme [15]. Total imaging time is ~12 h. After acquisition, the images were analyzed using the FSL software package (FMRIB, Oxford UK), with was used to create fractional anisotropy (FA), mean diffusivity (MD), axial diffusivity (AD), and radial diffusivity (RD) maps for each of the brains used in this study.

#### MRI registration and analysis

To visualize and compare any changes in the mouse brains, the anatomical images (or the *b* = 0 s/mm^2^ images from DTI) are linearly (6 followed by 12 parameters) and non-linearly registered together. Registrations were performed with a combination of mni_autoreg tools [16] and advanced normalization tools [17, 18]. After registration, all scans can be resampled with the appropriate transforms and averaged to create a population atlas representing the average anatomy of the study sample. Note that the 40um anatomical images and the *b* = 0 s/mm^2^ DTI images are registered separately. The result of this registration is to have all images deformed into alignment with each other in an unbiased fashion.

For the volume measurements, this allows for the analysis of the deformations needed to take each individual mouse’s anatomy into this final atlas space, the goal being to model how the deformation fields relate to genotype [19–21]. The jacobian determinants of the deformation fields are then calculated as measures of volume at each voxel. For the diffusion measurements, the registration allows for the analysis of the intensity differences of all measures (FA, MD, AD, and RD) between genotypes. Significant volume changes and intensity differences can then be calculated by warping a pre-existing classified MRI atlas onto the population atlas, which allows for the volume or mean diffusion measure (FA, MD, AD, and RD) of 182 different segmented structures encompassing cortical lobes, large white matter structures (i.e., corpus callosum), ventricles, cerebellum, brain stem, and olfactory bulbs [22–24] to be assessed in all brains. Further, these measurements can be examined on a voxel-wise basis in order to localize the differences found within regions or across the brain. Multiple comparisons in this study were controlled for using the False Discovery Rate [25].

### Quantitative real-time PCR

Testes were collected from adult mice to analyze the transcriptional activity of Class II ERV. The collected tissues were immediately frozen in liquid nitrogen and stored at −80 ^o^C until use. Each sample was homogenized in 1 ml TRI REAGENT® (Molecular Research Center, Inc.) for RNA extraction following the manufacturer’s instructions. RNA was treated with DNase I (Promega) and purified again by phenol/chloroform. 2.5 μg of total RNA was used for reverse transcription with random primers by SuperScript® II (Invitrogen). The PCR reaction was performed with 2 μl of 1/10 diluted cDNA template, specific primer pairs, and Power SYBR Green Master Mix (Thermo Fisher) in StepOnePlus ^TM^ (Thermo Fisher). Relative expression was calculated after normalizing to the endogenous control gene, *Gapdh* (Supplementary Method and Material), by using the comparative Ct method.

Primers for quantitative PCR [26, 27]:

**Table:** 

Class II ERV	Primer sequence	
Name	Forward	Reverse
ETnI	GTTAAACCCGAGCGCTGGTTC	GCTATAAGGCCCAGAGAGAAATTTC
ETnIIα	CCAGC(C/T)(A/C)TTCTAACTCAATC	GCAGGGAGTAATCTATGTAAG
ETnIIβ	CCAGC(C/T(A/C)TTCTAACTCAATC	CATT(T/C)(G/A)TTAGT(C/T)AGGGGGTATTAAGTGAC
ETnIIγ	GAGTTGTTTCAGGCCAGAGGAGTAAGG	TACCATTGTCAAACACATTAATCATGAACC
MusD	GATTGGTGGAAGTTTAGCTAGCAT	TAGCATTCTCATAAGCCAATTGCAT
*IAP*	AAGCAGCAATCACCCACTTTGG	CAATCATTAGATG(T/C)GGCTGCCAAG
*LINE*	TCGACATGGAGCTGGTGAAA	TCGACATGGAGCTGGTGAAA

### Behavioral tests

#### Pup ultrasonic vocalization (USV)

Both male and female pups on postnatal day 8 (p8) were analyzed for their USV calls during a 5-minute separation from their mothers. Each pup was placed onto fluffy bottom bedding in a glass beaker and moved into a dark, soundproof box. The USV calls were recorded using an ultrasonic microphone connected to a pre-amplifier set at a 250 kHz sampling rate (UltraSoundGate 416H, Avisoft Bioacoustics). The acoustic data were digitalized by an Avisoft signal conditioner and recorded with Avisoft-Recorder software during the recording. The call number and types of the first 3 min of recording were analyzed manually according to the previous study [28].

#### Self-grooming

The subject was first put into a clean and transparent cage without bedding for 10-min habituation and followed by a 10-min recording from the side view. The light intensity in the cage was around 30 lux. The total grooming time was analyzed manually.

#### Marble burying test

A clean cage with 5 cm ALPHA-dri bedding (Shepherd Specialty Papers) was prepared, and 20 clear blue marbles were set on the bedding. Each subject was put into the cage for 30 min. At the end of the test, the subject was removed from the cage. The location of the marbles was photographed and classified into still (at the same position), moved (move away but not buried), half (~50% covered with bedding), and buried (>50% covered with bedding). The light intensity at the cage was ~30 lux.

#### Three-chamber social interaction test

The test arena is a rectangular three-chambered box divided by two Plexiglas walls with small square openings (5 × 3 cm), which allow the subject to enter each chamber (O’HARA& CO., LTD). Each chamber was 20 × 40 × 22 cm in size, and each of the two side chambers had a small wire cage in quadrant shape at the corner. The arena was illuminated at 20 lux. The subject was first put into the central chamber and allowed to explore the entire 3-chamber box for habituation freely. Followed by a 10-minute habituation, in the first testing section, an age-matched unfamiliar mouse (S1) of the same strain or an inanimate object of comparable size was put into the wire cages on either side. The position of the stranger mouse and object changed alternatively for each subject. In the second section of preference for social novelty, the inanimate subject was replaced with another unfamiliar mouse (S2). The subject mouse was allowed to explore the familiar stranger (S1) or novel stranger (S2). Each section was 10 min, and the movement of each subject was recorded from the top view to analyze the time spent in around the wire cage, which was used to indicate whether the subject preferred a social object (S1 vs. empty; 1st section) or a novel social object (S2 vs. S1; 2nd section).

#### Barnes maze

The circular open field was 1 m in diameter with 12 holes equally spaced around the perimeter (O’HARA& CO., LTD) and elevated 75 cm above the floor. A black Plexiglas escape box (17 × 13 × 7 cm), covered by a layer of clean bedding, was set below one of the holes. The position of the escape box and target site was fixed and randomized for each mouse. Four visual cues were hung around the maze. The subject was released from the center to explore the maze for three trials per day and 6 successive days. The movement of the subject was recorded and analyzed by video tracking software (Image BM, O’HARA& CO. [29, 30], LTD; https://ohara-time.co.jp/products/barnes-circular-maze/).

## Results

### BTBR/R and BTBR/J show substantial differences in their neuroanatomy, immune profiles, and microbiota composition

The appearance of the two BTBR strains was indistinguishable, except for the short-tailed phenotype due to a *T* (brachyury) gene mutation, which is still carried by the BTBR/R strain but dropped in BTBR/J (Supplementary Fig. S1a). The two BTBR strains are recognized by their distinctive black and tan (belly hair) coat color, distinguishing them from the B6 mice by postnatal day 7. Both strains show the characteristic of hair loss in mature adults (usually after 10 week-old), which are due to carrying the mutations at *a* (nonagouti) and *Itpr3* (tufted) genes, respectively. BTBR/R mice also show faster growth and larger body weight/size than B6 from the postnatal stage, as previously observed in BTBR/J [11].

Unexpectedly, we found that BTBR/R has an intact corpus callosum in both shortened- and normal-tailed mice (Supplementary Fig. S1b, S1c). Agenesis of corpus callosum (AgCC) was an important feature linking BTBR/J to human autism [31], which shows resemblance to features of reduced long-range connectivity in the autistic brain [32] and corresponds to the study in AgCC patients: one-third of them meet the diagnostic criteria for autism [33]. However, as more attention was paid to the etiology of autism, the validity of AgCC was weakened considerably. Surgical lesion of the corpus callosum at an early postnatal stage does not result in the core symptoms of autism in mice [34]. The role of the corpus callosum in interhemispheric connection also calls the behavioral abnormalities observed in BTBR/J mice into question. Therefore, we decided to comprehensively compare the key features associated with BTBR/J as an idiopathic autism model. Homozygous of *T* mutation (*T* ^t^/*T* ^t^) is known to be embryonic lethal since the *T* gene encodes a T-box transcription factor crucial for the developmental process [35]. To reveal the authentic differences between the two BTBR strains, by the breeding selection, we excluded the short tail BTBR/R (*T* ^+^/*T* ^t^) from the comparative analysis to avoid any unknown dosage effect of *T* mutation, which could complicate the comparison to BTBR/J (*T* ^+^/*T* ^+^).

**Fig. 1:**
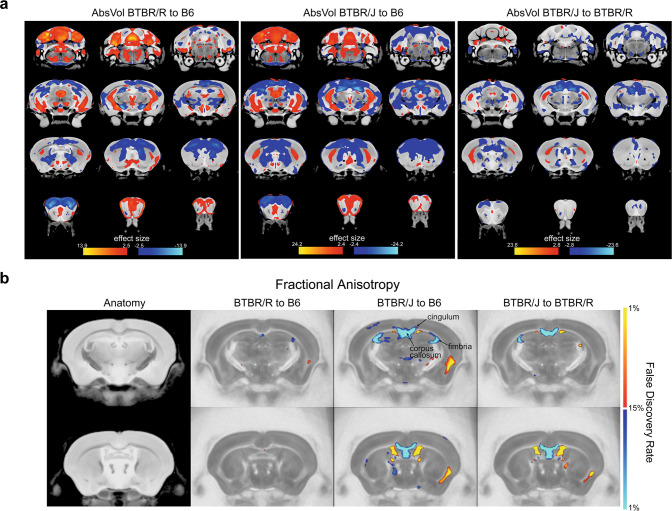
BTBR/R and BTBR/J showed substantial anatomic differences and had distinct white matter patterns, including AgCC. **a** Brain regions with significant differences in voxel-wise absolute volume. Comparisons of both strains to B6 and between each other were highlighted in red to show significantly larger or blue for significantly smaller volumes. All the highlighted changes have a significant FDR value of <1%. **b** DTI analysis for white matter pattern shown by FA (fractional anisotropy) difference. Comparisons of both strains to B6 and between each other were highlighted in red to show significantly larger or blue for significantly smaller volumes. All the highlighted changes have a significant FDR value of <1%. B6, *n* = 9; BTBR/R, *n* = 12; BTBR/J, *n* = 12.

First, we performed MRI analysis, including DTI, to scrutinize the neuroanatomic differences. The total brain volume did not differ among the two BTBR strains and B6 (Supplementary Fig. S1d). Of the 182 regions, 70 different areas from B6 were observed in both BTBR strains (Fig. 1a, Supplementary Table S1). These brain areas were enriched in the olfactory bulb, ventral hippocampus, cerebellum, primary and cingulate cortex, consistent with the previous studies in BTBR/J [36]. More interestingly, 33 regions were distinguishable between BTBR/R and BTBR/J, including enlarged amygdala in BTBR/R (Supplementary Table S2).

For brain connectivity, in addition to the corpus callosum, forebrain bundles known to be decreased in BTBR/J, including anterior commissure, fimbria, mammillothalamic tract, and stria medullaris [37], were normal in BTBR/R as compared to B6 (Fig. 1b, Supplementary Table S3). A recent study reported that the mutation in *Draxin*, encoding a chemorepulsive axon guidance molecule, leads to the forebrain bundle phenotypes in BTBR/J [38]. Of note, in BTBR/J, we found the same 8-bp deletion in *Draxin* exon 2, while an intact *Draxin* gene and normal expression were verified in BTBR/R (Supplementary Fig. S2d–f). In their axonal connectivity, BTBR/R mice are comparable to B6 mice, both regionally and voxelwise. Therefore, the *Draxin* mutation may prominently contribute to the white matter differences in BTBR/J and BTBR/R, providing the anatomic basis for the potential divergence in behavioral patterns.

We next compared the phenotype of immune dysregulation and the comorbid microbiota dysbiosis in the two strains. Of note, BTBR/R had higher expression of the pro-inflammatory cytokine, IL-6, in the brain but more moderate changes compared to B6 than BTBR/J in the peripheral immune system (Supplementary Fig. S3a–c). Both BTBR strains showed distinct microbiota compositions to B6 mice (Supplementary Figs. S3d,  S4). In contrast, BTBR/J mice had a more significant reduction of Lachnospiraceae/Ruminococcaceaem bacteria (Supplementary Table S4), whose lives are tightly associated with the regulatory T cells. This feature is consistent with the observations in the peripheral immune system and echoes our previous conclusion about the causality between immune dysregulation and gut dysbiosis [9].

### Accelerated de novo rate of CNV formation in BTBR strains

From the analysis above, it is apparent that substantial differences at multiple levels have accumulated between BTBR/R and BTBR/J within ~30-year separation, which raise a question about the underlying genomic mechanism. We first accessed the genetic background of the two BTBR strains by whole-genome SNP scanning using 154 markers across autosomal, sex chromosomes, and mitochondria DNA [39, 40] (Supplementary Table S5). Among them, 104 markers were similar to the 129 strain, and 46 were identical to the B6 strain, with only 4 markers differing between the 2 BTBR strains, suggesting similar genetic background to strain 129 as reported [41, 42]. To explore other genetic variants capable of impacting as many genes within 3 decades, we considered copy number variations (CNVs) as a possible mechanism for accelerating strain segregation. Although less frequent, studies have found a higher de novo CNV load in autistic individuals, suggesting a role in ASD pathology or susceptibility [2]. Systemic screening to directly compare the CNVs between the two BTBR strains was performed by array-based comparative genomic hybridization (SurePrint G3 Human CGH Microarray, 1 × 1M). Fifty-seven differential CNVs ranging from several Kb to hundreds of Kb were identified between BTBR/R and BTBR/J, which should include (i) BTBR/J-specific CNVs, (ii) BTBR/R-specific CNVs, and (iii) shared CNVs with different copy number within the two strains (Table 1). Twenty-five of 57 CNVs contained non-coding regions and the others contained genes related to neuronal function, human autism, and immunity. Interestingly, by examining the differential CNVs with more than four copies (seg.mean>2) between BTBR/R and BTBR/J, we found CNV hotspots concentrated either in the intergenic region or paralogous gene families. Considering the CNV hotspots fell within intergenic regions and paralogous gene families, this is consistent with the non-random distribution of transposable elements in the genome. These known selfish elements have evolved mechanisms to target specific loci in the host genome, which is a less deleterious effect on the host but still allows their propagation [43].

**Table 1:** Differential CNV between BTBR/R and BTBR/J.

chr	Gene in CNV	human ASD	loc.start	loc.end	num.mark	seg.mean
1	**sntg1**	8q11.21-q11.22	8620954	8622044	2	−2.4392918
1	**pkhd1**	6p12.3-p12.2	20494841	20498365	3	1.378600159
1	no gene	—	101333243	101335911	2	2.274856359
1	**Cntnap5b**	not found	101794048	101807488	8	1.623758453
1	**Cntnap5b**	not found	102200216	102201788	2	3.048533413
1	no gene	—	102445317	102455018	3	1.745673214
1	no gene	—	146799763	146818890	4	−1.14805835
2	no gene	—	53206274	53206705	2	1.286914944
2	no gene	—	129668401	129671450	2	−1.88419762
2	no gene	—	153855763	153857190	2	1.095239693
3	no gene	—	64661801	64664040	2	−2.45556925
4	no gene	—	6080457	6094385	5	−1.50117931
4	no gene	—	20008384	20011858	2	−2.23100691
4	**Skint6**	not found	112481921	112497729	5	2.381158058
4	**Skint5**	not found	113246661	113561150	63	1.497525185
4	**Hdac1**	1p35.2-p35.1	129204618	129205791	2	−1.31614791
4	**Col16a1**	—	129771943	129773080	2	−1.24074014
5	no gene	—	33522185	33523990	2	−2.42035681
5	**Otof**	not found	30733986	30735151	2	3.410291382
5	**Stx18**	4p16.3-p15.31	38485136	38487007	2	−1.97089994
5	no gene	—	38555107	38556695	2	−1.39222007
6	**Col28a1**	7p21.3	8041059	8050960	7	4.388931399
6	**Dnahc6**	not found	73152290	73153206	2	−1.33541509
6	**Klrb1c**	not found	128732657	128738467	4	2.07387971
6	**Klra17**	not found	129799173	129804403	2	3.382054993
6	**Klra18**	not found	129928851	129999391	12	3.366774124
6	**A630073D07Rik**	not found	132581120	132613035	19	3.515596503
7	**Luzp2**	11p14.3-p12	62093985	62096661	3	−2.55124078
7	**Lrrk1**	not found	73459398	73463240	4	−3.24153744
7	**AK031079**	not found	132458814	132463007	3	−2.84921773
8	**Csgalnact1**	8p22-p21.3	71029904	71030874	2	1.058698484
8	no gene	—	73469383	73475981	3	2.779125044
8	no gene	—	74542852	74562783	2	2.195462278
8	**Slc35e1**	19p13.12-q12	75008096	75010001	2	2.189815
9	**Opcml**	11q24.3-q25	28205943	28208060	2	1.484237473
9			28372011	28373436	2	1.554330156
9	**Ntm**	not found	29282071	29283655	2	1.600504956
11	**Nlrp1b**	not found	70993620	70996938	2	−1.19489754
11	**Nlrp1c**	not found	71076914	71086219	3	−2.07088997
11	no gene	—	83492037	83498686	6	−1.03777034
11	no gene	—	99730806	99732549	2	1.509151934
11	no gene	—	103371233	103389245	10	3.561439111
11	**St6galnac1**	17q25.1-q25.2	116603960	116626784	12	1.00853737
13	no gene	—	36065953	**36067910**	2	−1.80613231
14	**AK138521**	not found	45498060	45501402	3	1.432083202
14	**Ero1l**	not found	45906761	45908592	2	1.628409152
14	no gene	—	46361336	46364688	2	−1.34006579
14	no gene	—	68867248	68870499	2	−1.60482346
14	no gene	—	74441654	74450219	4	−2.53994622
14	no gene	—	75354943	75356406	2	−3.3727536
14	no gene	—	96499748	96502090	2	−2.34627781
15	**Apol10a**	not found	77310653	77370705	6	4.537421585
15	no gene	—	81137084	81138613	2	−2.1805136
17	**Vmn2r118**	not found	55758813	55760569	2	−1.52310403
X	no gene	—	73027553	73030265	2	3.808590561
X	no gene	—	132892550	132900847	2	−1.18194202
X	**Mid1**	Xp22.2	166409011	166428292	13	3.030595001
		Xp22.33-p22.2				

Another intriguing observation was that BTBR/R and BTBR/J have different copies of the CNV-carrying HDAC1 gene (Table 1, chr4: 129204618-129205791), suggesting different expression levels of HDAC1 in the two BTBR strains. In our previous study, we found that a disturbed epigenetic mechanism mediated by HDAC1 affects the development of microglia and hematopoietic stem cell (HSC), which leads respectively to immune dysregulation in the brain and the peripheral immune system of BTBR/R mice [9]. Since microglia and HSC have different epigenetic demands for their development, the varying levels of HDAC1 in BTBR/R and BTBR/J may account for the divergent immune phenotype in the two BTBR strains.

The average CNV accumulation rate between related strain pairs is 0.37 CNVs/ year of separation [44]. For the case of 129S1/SvImJ and 129 × 1/SvJ, which were separated for 55 years and had a genetic background close to BTBR, it is ~0.6 CNV/ per year. Intriguingly, the accumulation rate of BTBR/J and BTBR/R is 1.76 CNV per year (58 CNVs/33years: from 1982 to 2015, the year of our aCGH analysis), about 3-fold and 5-fold higher than the 129 strains and the average rate, respectively.

### Differential CNV between two BTBR strains are enriched for the sequence of the long terminal repeat (LTR)

**Fig. 2:**
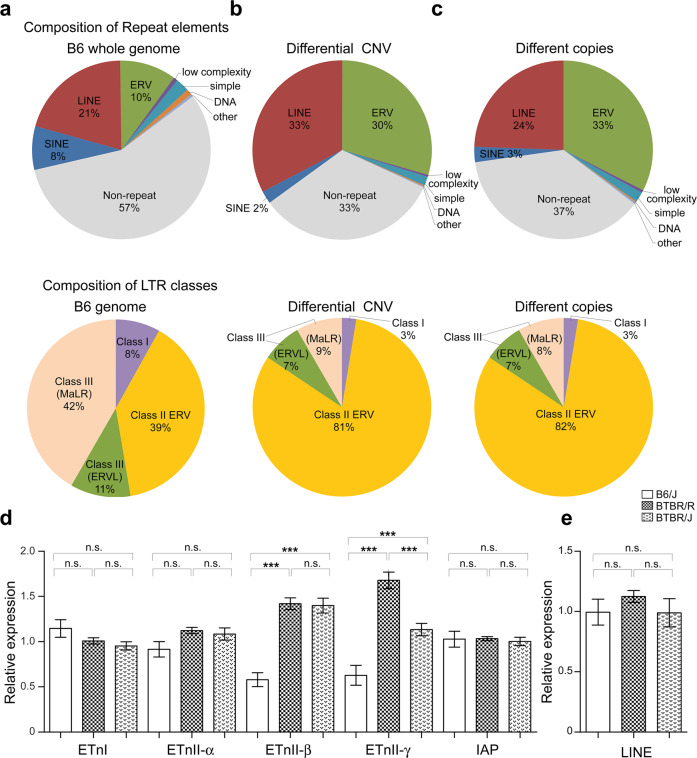
Endogenous retrovirus sequences are enriched in the differential CNV with increased transcriptional activity in BTBR strains. **a**–**c** top The composition of repeat elements in the B6 whole genome, the differential CNV between two BTBR strains, and the shared CNV of different copies were analyzed. **a**–**c**, bottom The composition of three classes of LTR endogenous retrovirus in the B6 whole genome, the differential CNV between two BTBR strains, and shared CNV of different copies. **d** The transcriptional activity of Class II Endogenous Retrovirus was analyzed in the testis germ cell in three strains. B6, *n* = 15; BTBR/R, *n* = 15; BTBR/J, *n* = 15. ETnI, one-way ANOVA (effect of genotype), *F*_2,42_ = 2.326, *P* = 0.1101; ETnII-α, one-way ANOVA (effect of genotype), *F*_2,42_ = 2.803, *P* = 0.0720; ETnII-β, one-way ANOVA (effect of genotype), *F*_2,42_ = 40.24, *P* < 0.001; ETnII-γ, one-way ANOVA (effect of genotype), *F*_2,42_ = 32.88, *P* < 0.001; IAP, one-way ANOVA (effect of genotype), *F*_2,42_ = 0.07889, *P* = 0.9243. **e** The transcriptional activity of non-LTR retroelement, LINE in testis germ cells in three strains. B6, *n* = 7; BTBR/R, *n* = 7; BTBR/J, *n* = 7. One-way ANOVA (effect of genotype), *F*_2,18_ = 0.6370, *P* = 0.5404. Data shown are mean (±S.E.M.) for each strain and analyzed by one-way ANOVA followed by Tukey’s multiple comparison test, ***, *p* < 0.001; **, *p* < 0.01; *, *p* < 0.05; n.s., not significant).

To investigate the mechanism of this accelerated CNV generation in BTBR strains, the sequence features of the 57 differential CNVs were analyzed based on the database of UCSC Genome Browser using NCBI37/mm9 Assembly. The repeat sequence of the long terminal repeat (LTR) transposon, also known as endogenous retrovirus (ERV), was enriched threefold (30%) (Fig. 2b, top) in the differential CNV as compared to the ERV sequence in the B6 whole genome, which is only 10% (Fig. 2a, top) [45]. BTBR/R gDNA was used as the reference genome for Fig. 2b. The prevalent mechanism of CNV formation is meiotic recombination between highly similar duplicated sequences, known as non-allelic homologous recombination (NAHR). Retrotransposons within the genome, including LTR and long interspersed element (LINE), can serve as seeds for NAHR to facilitate CNV formation [46, 47].

To further investigate the involvement of ERV in the accelerated CNV accumulation in the BTBR strain, we next compared the repeat sequence in the shared CNVs with different copy numbers within the two strains, which suggest potential hotspots for CNV formation. To identify the shared CNVs with different copy numbers, a second aCGH analysis was performed by comparing the genome of BTBR/R or BTBR/J to the B6 strain as a reference (Supplementary Table S6). These shared CNVs of different copies showed similar ERV composition of 33% in their sequences (Fig. 2c, top), which further confirmed the enrichment of ERV repeats in newly generated CNV. B6 gDNA was used as the reference genome for Fig. 2c.

Phylogenetic analysis based on the sequence similarity of reverse transcriptase has groups ERVs into three classes [48]. Therefore, we further dissected the ERV composition in the differential CNVs and found an increased ratio of Class II ERV, which includes several transcriptionally active members [49] (Fig. 2a–c, bottom). Active ERV can be transcribed and translated by the host machinery. The resulting mRNA can be reversely transcribed to cDNA and integrated back into the host genome, generating a new copy of ERV. We hypothesized that the accumulation of ERV copies in the genome would increase the chance of NAHR and accelerate the generation of CNV. We compared the transcriptional activity of different ERV families between B6 and BTBR strains to verify this possibility. Considering germline transmission is necessary for the accumulation of CNV, ERV expression was analyzed in germ cells, such as testis. By checking the known active members of Class II ERV [49], the results showed significantly higher activities of ETnII-β and ETnII-γ in both BTBR strains (Fig. 2d), suggesting the source for enriched Class II ERV repeats. We also found comparable expression levels of non-LTR retrotransposons, LINE, among three strains (Fig. 2e), suggesting a specific alteration in the epigenetic silencing mechanism against ERV in BTBR strains [50].

### Embryonic ERV activation evades integrated stress response and leads to global transcriptome changes across multiple cell types

**Fig. 3:**
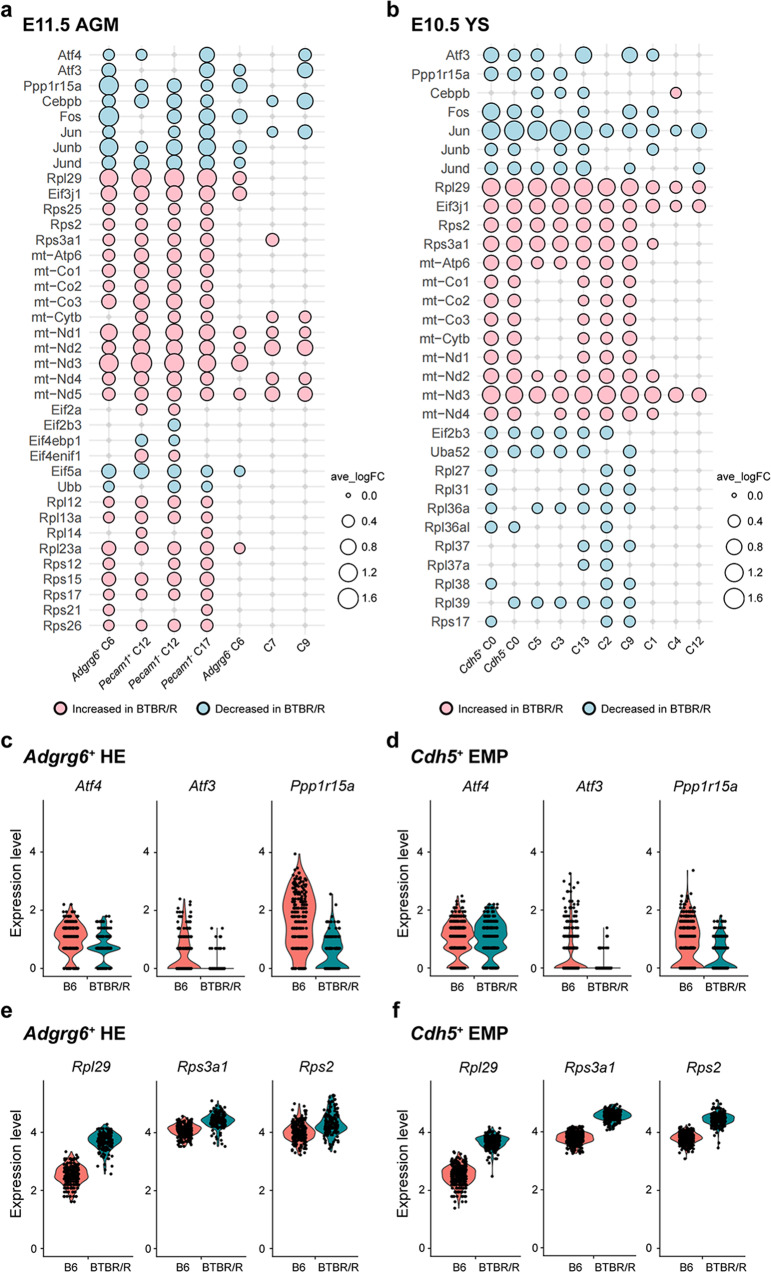
The single-cell RNA-seq of E11.5 AGM and E10.5 YS shows that the global transcriptomic changes encoding reduced ISR activation, IRES-mediated translation, and increased energy production in BTBR/R mice. **a** Bubble plot of DEG in AGM cell clusters. Progenitor cells across definitive hematopoiesis include *Adgrg6*^+^ hemogenic endothelium (HE) (cluster 6, C6), *Pecam1*^+^ pro-hematopoietic stem cells (ProHSC) (cluster 12, C12), *Pecam1*^-^ pre-hematopoietic stem cells type I (PreHSC I) (C12), pre-hematopoietic stem cells type II (PreHSC II) (cluster 17, C17); differentiated cell type includes *Adgrg6*^-^ HE (C6), cluster 7 and cluster 9 (See Supplementary Fig. 5 for cell clustering by UMAP). **b** Bubble plot of DEG in YS cell clusters indicated widespread changes centering on the viral infection process. Progenitor cells across definitive hematopoiesis include *Cdh5*^+^ erythro-myeloid progenitor (EMP) (cluster 0, C0), *Cdh5*^-^ EMP (C0), erythroid progenitors (EP) (cluster 3, C3), myeloid progenitors (MP) (cluster 5, C5); differentiated cell type includes hematopoietic cell (C3), microglia (cluster 13, C13), cluster 1, cluster 2, cluster 4 and cluster 9. All the DEGs had an FDR value of <0.05 with an average log fold-change (avg_ln FC) ≥ 0.25. Genes increased or decreased in BTBR/R compared to B6 were shown in pink or light blue, respectively. **c** The expression of key ISR mediator, *Atf4* and its downstream effectors, *Atf3* and *Ppp1r15a* in AGM *Adgrg6*^+^ HE. *Atf4*, *p* < 0.0001; *Atf3*, *p* < 0.0001; *Ppp1r15a*, *p* < 0.0001). **d** The expression of key ISR mediator, *Atf4* an**d** its downstream effectors, *Atf3* and *PPP1r15a* in YS *Cdh5*^+^ EMP. *Atf4*, *non-significant*; *Atf3*, *p* < 0.0001^;^ *Ppp1r15a*, *p* < 0.0001). **e** The expression of Ribosomal protein facilitating virus infection in AGM *Adgrg6*^+^ HE. *Rpl29*, *p* < 0.0001; *Rps3a1*, *p* < 0.0001; *Rps2*, *p* < 0.0001). **f** The expression of Ribosomal protein facilitating virus infection in YS *Cdh5*^+^ EMP. *Rpl29*, *p* < 0.0001; *Rps3a1*, *p* < 0.0001; *Rps2*, *p* < 0.0001).

ERVs are remnants of ancient retroviral infection; therefore, active ERV in BTBR mice seems analogous to viral infection. A recent study in the polyI:C model of maternal immune activation (MIA) suggests double-stranded RNA virus infection activates the integrated stress response (ISR), disrupting protein synthesis in the fetal brain [51]. In addition to germ cells, a previous study also demonstrated increased expression of Class II ERV in BTBR/J embryos [49], which prompted us to verify the impact of active ERV during developmental stages. By re-accessing our previous single-cell transcriptome data sets of E11.5 embryonic aorta-gonad-mesonephros (AGM) and E10.5 yolk sac (YS) in BTBR/R [9] (Supplementary Fig. S5a, b), we analyzed multiple cell types including progenitor cells across definitive hematopoiesis to evaluate the effect of ubiquitous ERV existence (Supplementary Fig. S5c). After graph-based clustering with UMAP and cell marker identification, we set a false discovery rate value of <0.05 with an average log fold-change (avg_ln FC) ≥ 0.25 to define the differentially expressed genes (DEGs) between B6 and BTBR/R (Fig. 3a, b).

To fight against the stress from virus infection, the host cells have evolved defensive mechanisms, such as ISR, which leads to global attenuation of cap-dependent translation to slow down virus invasion. On the other hand, some viruses have counteracting strategies against ISR [52] and hijack ribosomes via their internal ribosome entry site (IRES) [53], which restricts host gene expression of immune response genes and selectively produces viral components.

It is interesting to find that the key mediator of the ISR pathway, Atf4, and its downstream effector, Atf3 and Ppp1r15a, were significantly reduced in multiple cell types in both AGM and YS of BTBR/R mice, particularly in the progenitor cell types, in which ERVs are known to be more active [54, 55] (Fig. 3c, d). Of note, the interacting partners of Atf4, AP-1 transcription factors, which modulate Atf4 selectivity by forming heterodimers, were also profoundly reduced in the BTBR mice. Similar to the observation in herpes virus (a retrovirus)-infected cells [56, 57], ribosomal proteins (RPs) mRNA facilitate viral propagation were upregulated in BTBR/R mice, including Rpl29, the molecular signature of viral infection [58]; Rps3a1 and Rps2, which interact with viral IRES [59] (Fig. 3e, f); Eif3j, which is required for the assembly of the translation pre-initiation complex (PIC) at IRES to facilitate viral gene expression [60]. We also observed the extensive upregulation of mitochondrial subunits responsible for oxidative phosphorylation, suggesting increased energy production in ERV active cells (Supplementary Fig. S5e, f). Cap-dependent protein synthesis was significantly suppressed in YS-derived cells, with decreased expression levels of the eIF2 complex subunit (formation of 43S PIC at 5′ capped mRNA) and RPs of 60S ribosomal subunits; however, this effect of ISR seemed to be compromised in AGM-derived cells, in which numerous RPs of large and small ribosomal subunits were increased. Taken together with the profound decrease of Atf4 in AGM cells, these results suggest the potential difference of ERV activity in different cell types and a viral mechanism to facilitate viral protein expression but inhibit host translation simultaneously [57, 61]. These widespread changes are similar to the infection process of exogenous retrovirus by evading the host defense mechanism and hijacking host translation machinery [62, 63]. We also verified the trace of ERV infection in BTBR/J by checking *Rpl29* expression in testis (Supplementary Fig. S5g), in which increased ERV activity in the two BTBR strains has been confirmed. Increased *Rpl29* expression in BTBR/J but less level than BTBR/R suggested the potential different ERV activity in the two BTBR strains.

To prevent the detrimental effects of ERV activation, the host has evolved epigenetic mechanisms, centering on histone modification and DNA methylation, to silence transcriptional expression and transposition of ERV [64]. A previous genome-wide siRNA screen found that the direct recruitment of sumoylation factors to Class II ERV sequence in the genome facilitates the assembly of chromatin modifiers to maintain a suppressive epigenetic environment [65]. Interestingly, sumoylation modification genes, including Sumo2, Ube2i, Sae1, and Uba2, were significantly reduced in AGM-derived precursors (Supplementary Fig. S5h). These results provide clues to investigate the potential involvement of impaired epigenetic mechanisms in silencing ERV expression in BTBR mice.

We hypothesize that active ERV activation accompanied by transposition could disperse more ERV copies throughout the genome [48] and therefore increase the chance of CNV formation, driving genome evolvement toward ASD susceptibility in the long term [4]. Meanwhile, the acute responses upon ERV activation provide a living model to study the effect of retroviral infection during embryonic development on autism pathogenesis.

### BTBR/R has severer core symptoms of autism but intact forebrain commissures, a potential substitute for BTBR/J as an idiopathic autism model

The substantial differences, including neuroanatomy, between BTBR/R and BTBR/J suggest the potential dissimilarity in behavioral outcomes. On the other hand, since the *Draxin* mutation has been verified as the genetic cause for AgCC in BTBR/J and *Draxin* knockout mice show abnormalities in anxiety, spatial learning, and socio-emotional behaviors [66] (personal communication), we wondered whether BTBR/R is a better autism model, as it lacks the side effects of *Draxin* mutation.

**Fig. 4:**
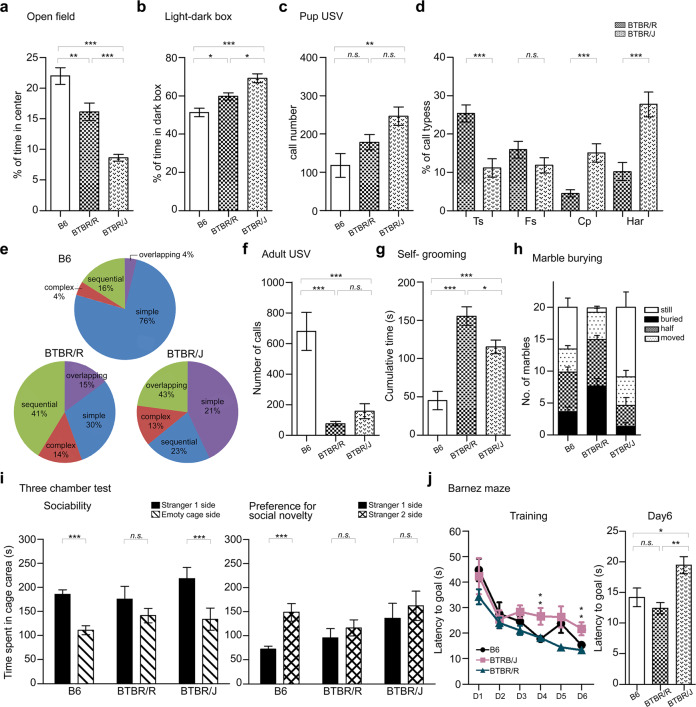
BTBR/R showed behavioral deficits and autistic phenotypes differently than BTBR/J. All the behavioral tests were analyzed in both BTBR strains and compared to B6. **a** Open field test in a 30-min section. The level of anxiety was accessed by the percentage of time spent in the center region. B6, *n* = 17; BTBR/R, *n* = 22; BTBR/J, *n* = 14. One-way ANOVA (effect of genotype), *F*_2,50_ = 22.89, *P* < 0.0001. **b** Light-dark box for 10 min. The level of anxiety was accessed by the time spent in the dark box. B6, *n* = 18; BTBR/R, *n* = 17; BTBR/J, *n* = 8. One-way ANOVA (effect of genotype), *F*_2,40_ = 13.43, *P* < 0.0001. **c** Pup USV calls. Total call number emitted by P8 pups within 3 min of maternal separation. B6, *n* = 17; BTBR/R, *n* = 26; BTBR/J, *n* = 28. One-way ANOVA (effect of genotype), *F*_2,68_ = 6.330, *P* = 0.0030. **d** Different preferences for call types of multiple syllables between two BTBR strains. Ts, two-syllable; Fs, frequency steps, Cp, composite; Har, harmonics. Unpaired t-test. **e** Call type usage summarized in pie charts for each strain. USV calls with single syllables were classified as “simple”. Calls of two-syllable and frequency steps were grouped as “sequential”; Calls of composite and harmonics were grouped as “overlapping” in the pie chart. **f** Adult male to female courtship calls within 5 min session. B6, *n* = 15; BTBR/R, *n* = 22; BTBR/J, *n* = 12. One-way ANOVA (effect of genotype), *F*_2,46_ = 21.89, *P* < 0.0001. **g** Repetitive behaviors analyzed by self-grooming in 10 min. B6, *n* = 14; BTBR/R, *n* = 22; BTBR/J, *n* = 14. One-way ANOVA (effect of genotype), *F*_2,47_ = 22.62, *P* < 0.0001. **h** Repetitive behaviors analyzed by marble burying in a 30 min section. The statistic result for the number of buried marbles. B6, *n* = 18; BTBR/R, *n* = 18; BTBR/J, *n* = 13. Still, one-way ANOVA (effect of genotype), *F*_2,46_ = 11.78, *P* < 0.0001; buried, one-way ANOVA (effect of genotype), *F*_2,46_ = 12.34, *P* < 0.0001; half, one-way ANOVA (effect of genotype), *F*_2,46_ = 5.208, *P* = 0.0091; moved, one-way ANOVA (effect of genotype), *F*_2,46_ = 0.3854, *P* = 0.6824. **i** Social interaction analyzed by three-chambered apparatus. The first 10-min section tested the sociability of the subject by analyzing its preference between a stranger mouse (stranger 1) and an empty cage. The second 10-min section tested the preference for social novelty by accessing the time spent on stranger 1 (more familiar) and stranger 2 mouse (novel). B6, *n* = 11; BTBR/R, *n* = 16; BTBR/J, *n* = 11. Unpaired t-test. **j** Spatial learning memory measured by Barnes maze. Left, the spatial performance profile of the 6-day training. B6, *n* = 18; BTBR/R, *n* = 21; BTBR/J, *n* = 13. D4, one-way ANOVA (effect of genotype), *F*_2,49_ = 5.983, *P* = 0.0047. D6, one-way ANOVA (effect of genotype), *F*_2,49_ = 6.782, *P* = 0.0025. Right, probe test on day 7. One-way ANOVA (effect of genotype), *F*_2,49_ = 7.390, *P* = 0.0016. B6, *n* = 11; BTBR/R, *n* = 16; BTBR/J, *n* = 11. Unpaired t-test. Data shown are mean (±S.E.M.) for each strain and analyzed by one-way ANOVA followed by Tukey’s multiple comparison test, ***, *p* < 0.001; **, *p* < 0.01; *, *p* < 0.05; n.s., not significant).

A behavioral test battery was performed to compare BTBR/R to BTBR/J using B6 as control. BTBR/R mice had reduced locomotor activity in the open field test (Supplementary Fig. S6a), but this was not observed in the light-dark box assay (Supplementary Fig. S6b). Anxiety, accessed by center time in the open field and light-dark transition, was elevated in both tests in BTBR/J (Fig. 4a, b). Motor learning ability was deficient in both strains, as assayed in the rotarod test (Supplementary Fig. S6c). Isolation-induced ultrasonic vocalization (USV) was first analyzed in pups on postnatal day 8. BTBR/J pups produced significantly more calls than the other strains (Fig. 4c). The vocalization repertoire was further analyzed and classified [28]. Interestingly, both BTBR strains preferred to use complicated calls of multiple syllables rather than short, single-syllable calls (classified as “simple”) usually emitted by B6 pups (Fig. 4d). Furthermore, among the multiple-syllable calls, BTBR/J emitted high levels of harmonics and composites (Har and Cp classified as “overlapping”) calls, as previously reported [6], while BTBR/R significantly used more two-syllable calls (Ts and Fs classified as “sequential”) (Fig. 4e). Female-induced USV in adult mice was analyzed, and the results also indicated that BTBR/R had deficits in social communication at levels comparable to BTBR/J (Fig. 4f). Taken together with the anatomic differences, it is interesting to note that brain regions involved in vocal communication circuitry, including M1, M2, anterodorsal striatum, and caudal periaqueductal gray (PAG), were all significantly different from B6 in both BTBR strains [67].

For repetitive behaviors, BTBR/R spent ~30% more time on self-grooming than BTBR/J mice (left, Fig. 4g). In the marble-burying test, BTBR/R showed an even stronger repetitive behavioral phenotype with more than 75% of the marbles completely or half buried (Fig. 4h). In contrast, BTBR/J showed limited deficits in the assay. Social behaviors were analyzed by the three-chamber test, which accesses sociability and preference for the social novelty of the testing subjects in two sequential sections. BTBR/J mice spent more time interacting with a stranger mouse one (S1) over an inanimate object (empty) but failed to show preference toward a novel social stimulus (S2) (Fig. 4i). BTBR/R mice did not show any preference in their interaction time in either section, suggesting severer social deficits.

In the Y-maze, it was found that both BTBR strains had impairments in working memory (Supplementary Fig. S6d), similar to the observation in autistic patients [68, 69]. Intriguingly, when hippocampus-dependent memory was analyzed by the Barnes maze, BTBR/R tended to learn the task faster than B6 and had similar latency to the goal at the end of the training, while BTBR/J demonstrated poor performance across the entire training period. Consistent with anatomic results, these results indicate that spatial learning/memory was normal in BTBR/R but impaired in BTBR/J (Fig. 4j). Of note, BTBR/R had severer core symptoms of autism, including repetitive behaviors and social deficits but milder associated symptoms, such as anxiety, USV communication, showing resemblance to autistic patients with higher function. The intact hippocampus-dependent episodic memory in BTBR/R mice also partially disassociates the etiology of autism from the mechanism underlying intellectual disability, providing excellent access to the core common symptoms of autism.

## Discussion

**Fig. 5:**
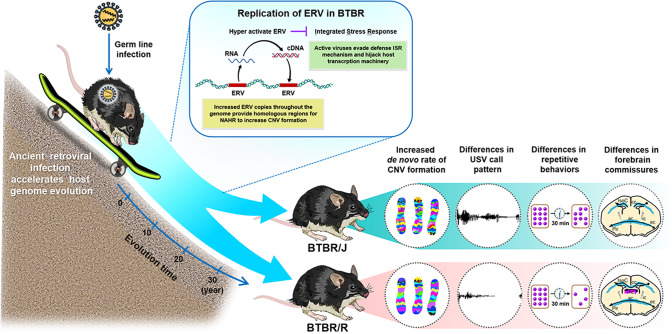
New advance in the old model: the impact of ERV activation on autism susceptibility by driving host genome evolution and invading ISR pathway. Hyper-activation of ancient retroviral infection accelerates host genome evolution toward ASD susceptibility by increasing the chance of CNV formation. The accumulated genetic variations lead to the divergence of autistic-like behaviors in both BTBR strains. Active ERV also recapitulates the viral infection process of ISR pathway invasion and IRES-mediated translation, which changes the global transcriptome during embryonic development in BTBR mice. BTBR/R has severer core symptoms of autism and wildtype Draxin expression, which suggests BTBR/R is a valid autism model with unaffected forebrain bundles.

BTBR/J has been recognized as one of the most validated mouse models of autism due to its robust behavioral analogies to the core symptoms in clinical diagnosis [70]. Here we reported a sister strain, BTBR/R, which shows comparable phenotypes of immune dysregulation and microbiota dysbiosis to BTBR/J while exhibiting more prominent autism core symptoms and normal hippocampus-dependent memory. We found the genetic footprint of ERV in the de novo CNV and transcriptionally active ERV in the two BTBR strains, which may help accelerate the segregation between the two strains (Fig. 5). ERVs are remnants of ancient retrovirus infection in the germline. We hypothesize that during the long journey of virus-host coevolution, unknown mutations occurred in the ancient BTBR founders, which disrupted the host’s epigenetic suppression on ERVs activity. After establishing segregated colonies, a chance mutation of an 8-bp deletion resulted in a premature stop codon in the *Draxin* exon of BTBR/J mice. This further added the phenotypes of hippocampus shrinkage and reduced forebrain bundles to this sister strain. This idea corresponds to the findings in the LP/J strain, which is genetically closest to BTBR/J but has an intact corpus callosum, and showed deficits in social and repetitive behaviors [34].

Interestingly, most of the differential CNVs between the two BTBR strains are found to have more than one copy of duplication or deletion (Seg. Mean >1 or <−1), suggesting recurrent CNVs generated by NAHR events. The distributions of highly homologous pairs of ERV elements are known to determine the susceptibility regions for recombination events [47]. One of the well-studied examples of ERV-mediated CNV formation is the recurrent deletion of Yq12.2, which is caused by intrachromosomal NAHR between human ERV elements (HERV-I) and leads to male infertility [71, 72]. Other clinical cases include the 1q41q42 deletion [73], the 8q13.3 deletion [74, 75] and the 4(4;18) (q35.1; q22.3) translocation [76]. A more recent study found a recurrent deletion of 3q13.2-q13.31 in nine individuals with the HERV sequences mapped at the breakpoint of this deletion. All nine individuals variably express cognitive delays and abnormal behavior, and three of them are diagnosed with ASD [77]. An independent study reported another case about a patient with ASD carrying the same microdeletion [78]. Therefore, this clinical evidence supports our hypothesis that active ERV associated with retrotransposition can disperse ERV copies throughout the genome and increase the chance of CNV formation in the two BTBR strains. Of note, enhanced HERV expressions in individuals with ASD have been reported by multiple groups [79, 80]. Taken together with the demonstrated roles of ERV in CNV formation, our observation of the two BTBR strains provides a vivid model to describe how the genome evolves toward ASD susceptibility.

For a successful infection, viruses must employ the host cell’s protein synthesis machinery to restrict immune response and produce viral components for their survival and spread. On the other hand, the host also has evolved defensive mechanisms, such as ISR, to downregulate RNA translation to slow down the viral invasion. This phenomenon is observed in the Poly(I:C)-induced MIA model, suggesting the synthetic double-stranded RNA might directly activate the ISR pathways in the cells of fetal brains [51]. On the other hand, human cytomegaloviruses and herpes viruses have established mechanisms to suppress host mRNA translation while upregulating ribosomal protein expression [56, 57, 61, 81], which are similar to the observation of active ERV in BTBR mice.

In addition to being the building blocks for human (8%) and mouse (10%) genomes, the 5′ and 3′ long-terminal repeats (LTRs) of ERV are enriched with transcription factor-binding site, which serve as critical regulatory elements to control its own activity and the expression of nearby host genes [82, 83]. It has been demonstrated that ERVs co-evolve with the host genome by providing their LTRs as alternative promoters for the stage- or tissue-specific expression of host genes [84, 85]. Accumulating studies suggest the involvement of active ERVs in mammalian development, including embryogenesis, cell differentiation, and germ cells, as well as roles in cancer [86] and neurological diseases [87, 88] when over-activated. Therefore, ERV activity must be tightly controlled to avoid aberrant gene expression and ensure host genome stability. Formation of heterochromatin structure by recruiting the histone modifying complex is the prominent mechanism of ERV silencing [64]. It is intriguing to note that the transcriptional activity of ERV is also enhanced in another autism mouse model induced by prenatal injection of high-dose valproic acid (VPA) [49], which is a potent HDAC inhibitor. Taken together, the findings among idiopathic models of ASD, including environmental (MIA- and VPA induced) and genetic (BTBR) ones, infection (either exogenous or endogenous) modulating the translational machinery centering on ISR and epigenetic dynamics seem to be entangled in the pathogenesis of autism, particularly in the autism subtype of immune dysregulation.

Escaping from the epigenetic silencing, active ERV in germ cells accelerates de novo CNV formation, which increases genome instability and makes BTBR strains a still evolving multiple-loci model of autism [4]. Meanwhile, active ERV during embryonic development invades the ISR pathway and alters the global gene expression profiles, which provides the second hit to the pathogenesis of autism in the BTBR strains. In conclusion, this study unravels the idiopathic etiology of the BTBR strain by suggesting it as a superimposed model of multiple genetic mechanisms and virus infection. Targeting the enhanced ERV activity or its infection process in BTBR mice will be the next step in considering the therapeutic strategies for ASD of immune-dysregulated subtypes. It will also be essential to examine the status of ISR in VPA-induced autism mice and to disclose how environmental factors affect ERV activity.

### Supplementary information

Supplemental text and figures

Supplemental table 1

Supplemental table 2

Supplemental table 3

Supplemental table 4

Supplemental table 5

Supplemental table 6
