# Supplementary material for: An old model with new insights: endogenous retroviruses drive the evolvement toward ASD susceptibility and hijack transcription machinery during development
Source: Mol Psychiatry. 2023 Mar 7;28(5):1932–45. doi: 10.1038/s41380-023-01999-z (PMC10575786; doi:10.1038/s41380-023-01999-z)
Supplement: Supplementary file 1 — Supplemental text and figures [file 41380_2023_1999_MOESM1_ESM.docx]

**Supplementary Information**

**An old model with new insights: endogenous retroviruses drive the evolvement toward ASD susceptibility and hijacks transcription machinery during development**

Chia-Wen Lin, Jacob Ellegood, Kota Tamada, Ikuo Miura, Mikiko Konda, Kozue Takeshita, Koji Atarashi, Jason P Lerch, Shigeharu Wakana, Thomas J. McHugh^2^ and Toru Takumi^*^

*Corresponding author. Email: [takumit@med.kobe-u.ac.jp](mailto:takumit@med.kobe-u.ac.jp)

This file includes Supplementary Methods and Materials, Supplementary results, Supplementary Table 1-6 and Supplementary Fig. 1-6, and Supplementary references.

**Supplemental Methods and Materials**

**Nissl stain**

Mice were anesthetized and perfused with PBS, followed by 4% paraformaldehyde/PBS. The brains were removed from the skull and had an overnight post-fixation at 4^o^C. Coronal sections were sliced at a thickness of 50 μm by vibratome (VT1200S, Leica Microsystems). Brain sections were mounted to the glass slides and dried in air. The slides were put into 1:1 alcohol/chloroform overnight to remove fat and rehydrated from 100%, 95% alcohol to distilled water. Slides were transferred into 0.1% cresyl violet solution for 5 minutes and rinsed quickly in distilled water. Followed by 95% ethyl alcohol for 2 minutes, the slides were dehydrated in 100% alcohol for 5 minutes (twice), xylene for 5 minutes (twice), and mounted with a permanent mounting medium (Permount® Fisher, Fisher Scientific Inc.)

**Draxin cloning**

Fresh RNA was prepared from the P8 brain of BTBR/R and BTBR/J. cDNA of Draxin was reverse transcribed using primer pairs for the coding region (Fw, CACCCGTGCACCTCTCC; Rv, AGGACTGGATGATCTGGGCT) and further amplified by KOD FX Neo (TOYOBO). PCR product was electrophoresed, and DNA at the predicted size was eluted from the gel for TA cloning (TOPO® TA Cloning Kit, Invitrogen). After colony PCR, positive clones were amplified for Miniprep DNA purification and sequenced using Sp6 primer.

**Quantitative real-time PCR**

To analyze cytokine expression, P5 mice were perfused with cold PBS to reduce the contamination from blood. Primers for quantitative PCR[^1^](#_ENREF_1):

|  | Primer sequence | | Amplicon length |
| --- | --- | --- | --- |
| Gene name | Forward | Reverse | (bp) |
| *Il6* | CCTACCCCAATTTCCAATGCT | GAATTGGATGGTCTTGGTCCTTA | 81 |
| *Tnf* | TCTTCTCATTCCTGCTTGTGG | GGTCTGGGCCATAGAACTGA | 128 |
| *Il1b* | GTCGCTCAGGGTCACAAGAAA | CATCAGAGGCAAGGAGGAAAAC | 74 |
| *Ccl2* | GGCTCAGCCAGATGCAGTTAA | CCAGCCTACTCATTGGGATCAT | 80 |
| *Ifnb* | CCTACAGGGCGGACTTCAAG | TGGATGGCAAAGGCAGTGT | 76 |
| *Ccl5* | GCAAGTGCTCCAATCTTGCA | CTTCTCTGGGTTGGCACACA | 71 |
| *Il10* | AGGCAGCCTTGCAGAAAAGA | AGTAAGAGCAGGCAGCATAGCA | 68 |
| *Tgfb* | TCGACATGGAGCTGGTGAAA | CTGGCGAGCCTTAGTTTGGA | 74 |
| *Gapdh* | GACGGCCGCATCTTCTTGT | CACACCGACCTTCACCATTTT | 65 |

**Flow cytometry analysis**

Inguinal lymph nodes and spleens were collected and dissociated in PBS. Spleen cell suspension was treated with 1x ACK lysing buffer to remove red blood cells. 1 x 10^6^ cells per sample were stained with Zombie Red^TM^ (BioLegend) to label dead cells, followed by fixation and permeabilization by Foxp3/Transcription Factor Staining Buffer Kit (TONBO bioscience). The following antibodies were used to stain the cells: CD3, CD4, Foxp3, CD19, B220 (all from BioLegend). Flow cytometry was performed by cell sorter SH800 (Sony Corp.) and analyzed by SH800 software.

**16S rRNA metagenomic sequencing**

DNA extraction and 16S rRNA gene amplification were performed according to the previously reported method with minor modification. Cecal content of 10 mg was suspended in 20% glycerol/PBS containing 10 mM EDTA of 200 μl and digested by 15 mg/ml lysozyme (Sigma) at 37^o^C for 1 hour, 2000 U/ml Achromopeptidase (FUJIFILM Wako Pure Chemical Corporation) at 37^o^C for 30 minutes and 1 mg/ml Proteinase K (Merck) at 55^o^C for 1 hour, sequentially. Bacteria DNA was extracted by phenol/chloroform and precipitated with 3 M sodium acetate. DNA concentration and quality were checked by Qubit and electrophoresis. 100 ng of DNA was required for 16S rRNA gene sequencing. V1-V2 region of 16S rRNA gene was amplified with primer sets of universal primer (27Fmod and 338R) and 454 adaptor sequence with barcode by Ex Taq (Takara). Amplicons were purified by AMPur XP (Beckman Coulter) and quantified by using Quant-iT Picogreen dsDNA assay kit (Invitrogen) and TBS-380mini fluorometer (Turner Biosystems). The amplified DNA was applied to 454 GS Junior pyrosequencing using GS Junior Titanium emPCR Kit-Lib-L, GS Junior Titanium Sequencing Kit, and GS Junior Titanium PicoTiterPlate Kit (all Roche) according to the manufacturer’s instructions. Reads with average quality value (QV) <25 and those lacking primer sequences of both ends were excluded. After trimming off the primer sequence at both ends, 3000 reads per sample were randomly selected for operational taxonomic units (OTUs) clustering with a cutoff of 97% pairwise identity by UCLUST program v.5.2.32 (<http://www.drive5.com/>). Representative sequences of the generated OTUs were blasted to the public database of Ribosomal Database Project (RDP), COR[E (http://microbi](file:///C:\Users\Chiawen\Desktop\E%20(http:\microbi)ome.osu.edu/), and in-house databases constructed from genome sequences available in NCBI and HMP databases.

**Whole-genome SNP scanning**

Genomic DNA was extracted from the tails by using Blood & Cell Culture DNA Mini Kit (Qiagen). Genome-wide SNP genotyping was performed by using TaqMan Minor Groove Binder (MGB) system (Applied Biosystems). TaqMan MGB probe sets were designed for 154 SNPs between C57BL/6J and strain 129 covering autosomal, X, Y chromosome, and mitochondrial DNA (Table S5). Quantitative PCR was performed using TaqMan Genotyping Master Mix (Applied Biosystems) and SNPs were detected using ABI PRISM 7900HT Sequence Detection System (Applied Biosystems). Please refer to the following website of Japan Mouse Clinic. https://ja.brc.riken.jp/lab/jmc/mouse_clinic/en/assistive/index.html

**Behavioral test**

***Open field***

The open field apparatus of 50 cm (length) x 50 cm (width) was illuminated with an LED of 25 lux. Each mouse was put in the corner at the beginning and allowed to explore the field for 30 min. The movement was recorded from above and analyzed for total distance traveled and time spent in the center area, defined as a central square of 30 cm x 30 cm (O’HARA& CO., LTD).

***Light/dark transition***

The apparatus was a box (21 cm x 42 x 25 cm) divided into two parts of the same size with a sliding door in the partition (O’HARA& CO., LTD). The light side was illuminated at 250 lux. The subject was initially put in the light slide and allowed two compartments freely for 10 min. The image was recorded by a CCD camera set on the ceiling of both rooms and analyzed by Image LD software (O’HARA& CO., LTD) for the subject’s movement and transition number.

***Y maze***

The apparatus contained three arms crossed at 120 degrees. The maze arms were in V-shape with 40 cm in length, 12 cm in height, and 3 cm in the bottom. Each subject was put at the root of the arm and allowed to explore the maze for 10 mins. The number of entries and alterations were analyzed by Y-maze software (O’HARA& CO., LTD).

***Adult USV***

The courtship USV emitted by male mice of fourteen- to sixteen-week-old was analyzed. The individual male mouse was first put into the testing field (a white plastic beaker of 23 cm in diameter and 27 cm in height), which was kept in the dark and soundproof box for 10 min habituation. An unfamiliar female mouse (10-weeks-old) was then put into the same testing field and the emitted USV was recorded for 5 min. The recordings were transferred to SASLab Pro and fast Fourier transformed with USV amplitudes under 30 kHz filtered to generate spectrograms and the calculation of total call number[^2^](#_ENREF_2).

**Quantification and statistical analysis**

The sample sizes were not predetermined but referred to the previous study[^1^](#_ENREF_1)^,^ [^3^](#_ENREF_3). Animals for behavioral analysis and tissue/fecal samples were collected from different litters to ensure the reproducibility of the observed phenotypes. The samples of B6 and the BTBR strains were included/prepared at the same time for each batch of the experiment to reduce the batch effects. For cytokine expression profiles and ERV transcriptional activities analyzed by quantitative PCR, the individual sample/animal with a deviated expression of any one of the genes analyzed was excluded from the analysis. The investigator was blind to the subject identity when analyzing the results of self-grooming and marble burying experiments. For the rest of experiments, no blinding was done since the analysis was not done manually. Statistical analysis was conducted using GraphPad Prism 5 or 9. The observed number of α-diversity, including OTUs, Chao1, ACE, and Shannon’s indices, were shown as box plots with the line of media inside the box. Whiskers represent the lowest/highest values and dots indicate outliers with values that exceed the 1.5 interquartile range (IQR). The other bar graphs were presented as mean+ (or ±) s.e.m. DEGs identified in sc-RNA seq were presented as violin plots with individual data points shown. Statistical analysis performed include two-tailed Student’s t-test, Mann-Whitney U test (differential phyla, genera, and OTUs), PERMANOVA (unweighted/weighted UniFrac distance), Wilcoxon rank-sum test with Benjamini-Hochberg correction FDR<0.05 (DEGs), and One-way ANOVA with Tukey’s multiple comparison test as appropriate. *p* values were presented in the figure legends. The threshold for statistical significance was set at *p*≤ 0.05 with *, p < 0.05; **, p < 0.01; ***, p < 0.001.

**Supplemental results**

***Draxin,* one of the multiple genes involved in the inheritance of AgCC phenotypes**

To investigate the genetic causes for the differential corpus callosum phenotype (CC) between two strains, the inheritance pattern of CC was analyzed in the F1 hybrid from both strains. The length of CC was compared to BTBR/R of the same gender and age (Supplementary Fig. S2a). Milder CC reduction was observed in F1 males, while severer and more variable CC reduction was found in F1 females, particularly from BTBR/R**^♂^** x BTBR/J**^♀^**. This sexual dimorphism trait suggested that multiple genes might be involved in the differential CC phenotype in two strains (Supplementary Fig. S2b). The genetic basis of AgCC had been accessed by Quantitative Trait Loci (QTL) analysis in previous studies and revealed candidate genes on chromosome 4[^4^](#_ENREF_4). In this study, the expression levels of these candidate genes were further analyzed by qPCR in the brains of two BTBR strains (Supplementary Fig. S2c). Among the 13 genes, *Draxin*, a chemorepulsive axon guidance molecule, was dramatically decreased BTBR/J (Supplementary Fig. S2d). Subsequently, *Draxin* cDNAs of two BTBR strains were cloned and sequenced. *Draxin* from BTBR/J had an 8-bp deletion, which led to a premature stop codon in exon 2 (Supplementary Fig. S2e). The confirmed reduction of *Draxin* at the protein level in BTBR/J was shown in Supplementary Fig. S2f. *Draxin* knockout mice lack forebrain commissures, including the corpus callosum, hippocampal commissures, and anterior commissures[^5^](#_ENREF_5), and have reduced the size of the anterior hippocampus[^6^](#_ENREF_6). These anatomic abnormalities show a remarkable resemblance to BTBR/J, suggesting that the AgCC phenotype in BTBR/J is mainly contributed by *Draxin* mutation.

**BTBR/R shows similar systemic immune dysregulation and gut microbiota dysbiosis**

Despite the well-documented immune abnormalities in individuals with ASD, BTBR/J is one of the few animal models to recapitulate these phenotype[^7^](#_ENREF_7)^,^ [^8^](#_ENREF_8). Previous studies have reported changes in the lymphoid cell population in peripheral immune organs and increased expression of cytokines in the brain of juvenile BTBR/J[^9^](#_ENREF_9)^,^ [^10^](#_ENREF_10). Here, we analyzed the inflammatory status in postnatal brains of BTBR/R and compared it to B6 and BTBR/J. Intriguingly BTBR/R had higher expression levels of the pro-inflammatory cytokine, IL-6, than BTBR/J (Supplementary Fig. S3a). For the immune profiles in the peripheral system, BTBR/R showed similar patterns of reduced B cells and increased CD4^+^ T cells population in the spleen and lymph node, but they were moderate levels than BTBR/J Supplementary Fig. S3b, S3c).

In BTBR/J mice, the difference in microbiota composition from the B6 strain has been demonstrated at α- and β-diversities by 16S rRNA sequencing[^11^](#_ENREF_11)^,^ [^12^](#_ENREF_12). We further compared the microbiome among the three strains by our sequencing pipeline and found similar segregation in BTBR/R from B6 and differences between both strains (Supplementary Fig. S3d, S3e). Both BTBR strains showed similar changes in the phylum of Actinobacteria and TM7 and three major genera (Supplementary Fig. S4a, S4b). Furthermore, at the species level, we found that operational taxonomic units (OTUs) belonging to the family Lachnospiraceae and Ruminococcaceae (Clostridium Cluster XIVa and IV), which generate short-chain fatty acid (SCFA), particularly butyrate to maintain intestine health, were significantly reduced in the microbiome of BTBR strains (Table S3). The alteration was even more significant in BTBR/J mice since the difference occurred at the genus level, reflecting the higher Treg population in BTBR/J than BTBR/R. On the contrary, *Lactobacilli* bacteria were largely increased in both strains at genus and species level (Supplementary Fig. S4b, Table S3). These observations confirmed the altered balance of probiotic bacteria in the gut ecosystem of BTBR mice. A more noticeable change in the Treg population in BTBR/J correlated with a more significant reduction of Lachnospiraceae/ Ruminococcaceaem bacteria.

Considering the behavioral analysis result, abnormal wiring related to AgCC found in BTBR/J may not be the leading cause for autistic behaviors but may worsen USV and anxiety phenotypes. Higher brain inflammation seems to be correlated to severer autism-specific behaviors in BTBR/R, including self-grooming and social impairment. This result suggested BTBR/R as a better model to study immune-related ASD.

**Supplementary Figures**

**Fig. S1**

**
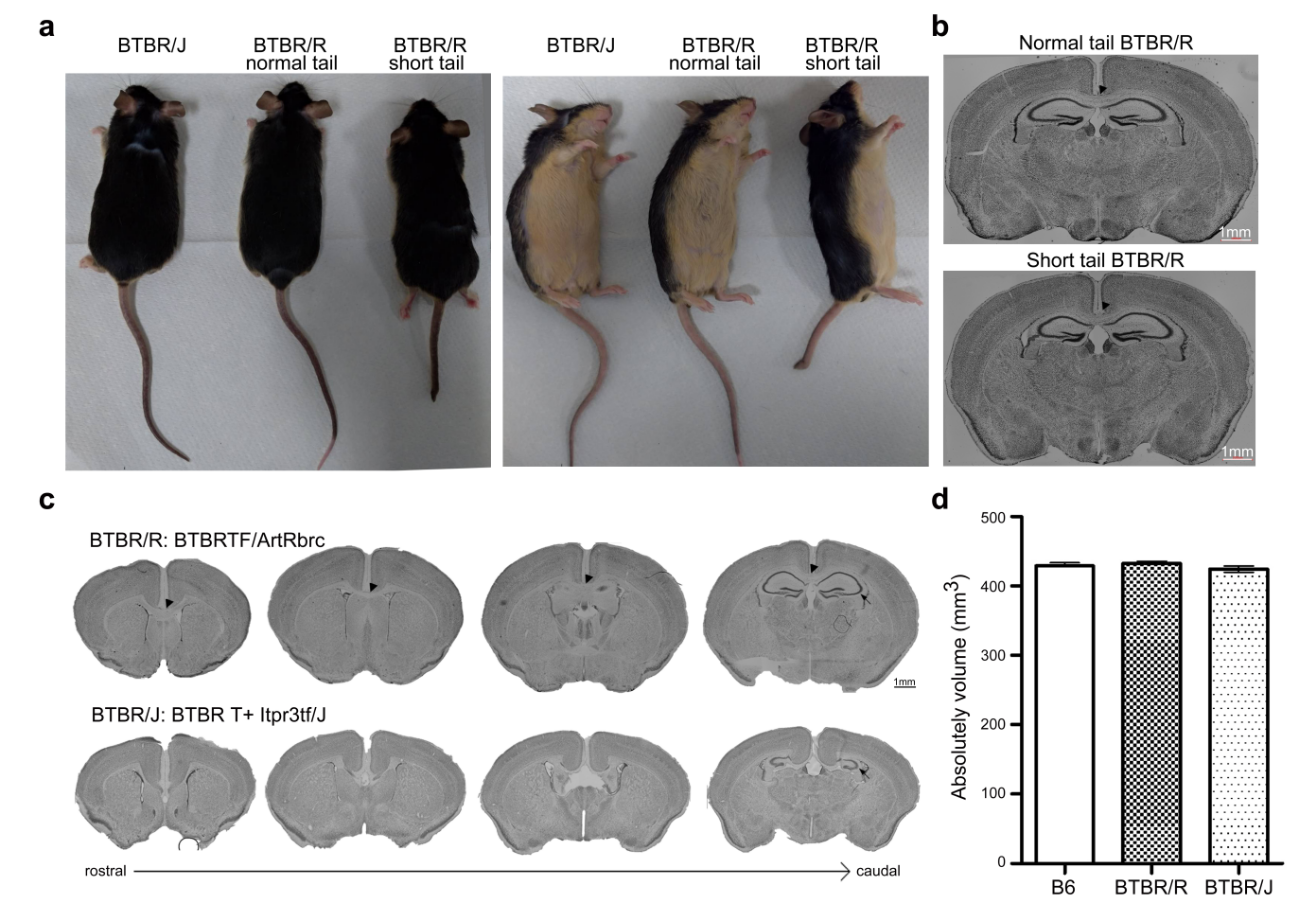
**

**Supplementary Fig. S1 BTBR/R and BTBR/J had distinct white matter patterns, including the AgCC phenotype. a** The appearance of BTBR/R and BTBR/J. **b** Intact corpus callosum in long-tailed and short-tailed BTBR/R of P25 mice. Arrowheads indicate corpus callosum. Scale bar: 1 mm. **c** Anatomic analysis by Nissl staining in the brain of 4-week-old mice. Top, BTBR/R; bottom, BTBR/J. Arrowheads indicate corpus callosum, which is intact in BTBR/R but agenesis in BTBR/J. Arrows indicate hippocampal structures. Scale bar: 1 mm. **d** The absolute volume of the total brain measured by MRI. B6, n=9; BTBR/R, n=12; BTBR/J, n=12. Data shown are the mean (±S.E.M.) for each strain. There is no statistically significant difference among three strains.

**Fig. S****2**

**
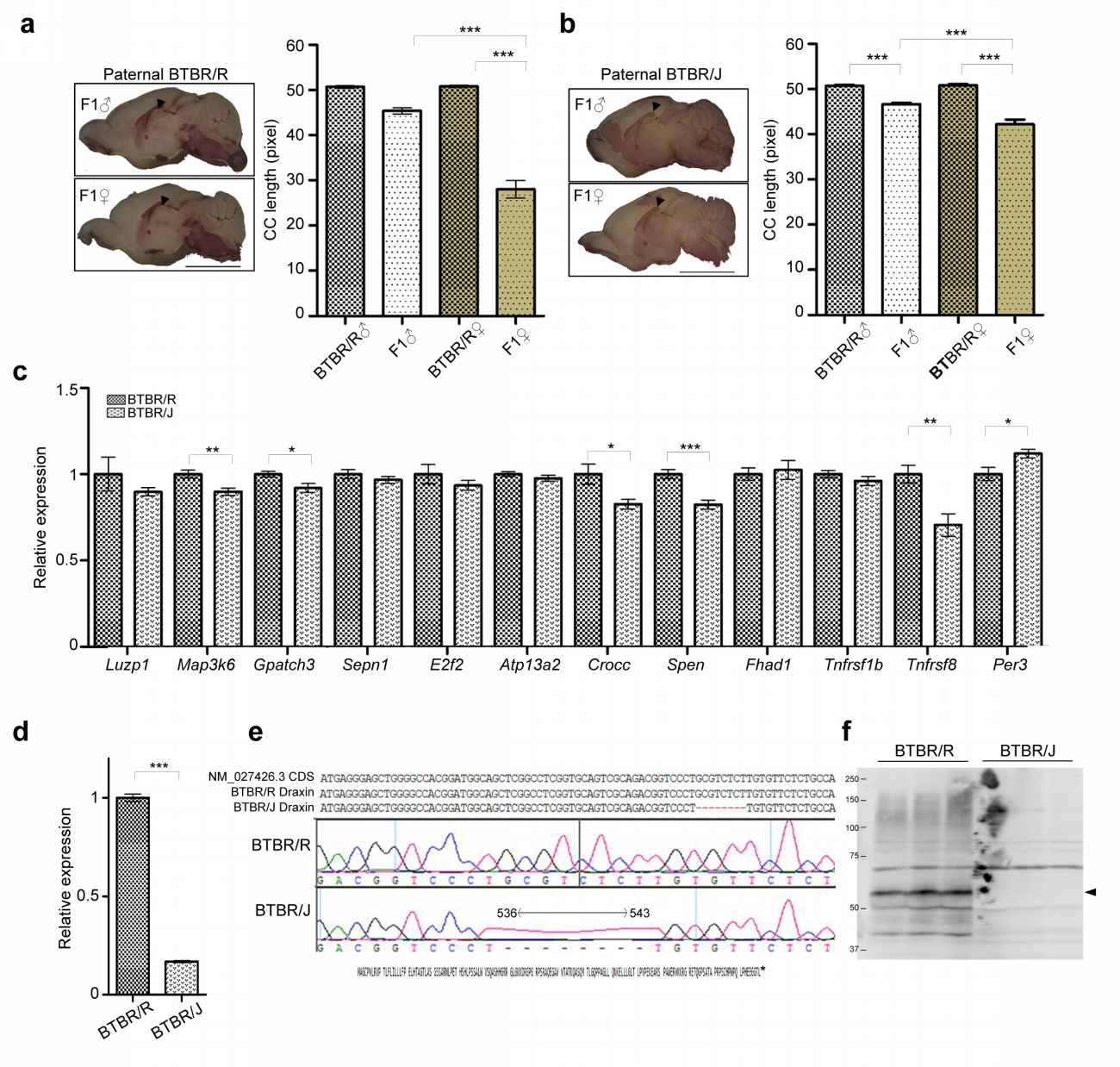
**

**Supplementary Fig. S2 BTBR/J carries a Draxin mutation, which leads to its AgCC phenotype.**  **a** The inheritance pattern of the corpus callosum in F1 hybrid of BTBR/R male and BTBR/J female. **b** The inheritance pattern of the corpus callosum in F1 hybrid of BTBR/J male and BTBR/R female. Arrowhead indicates corpus callosum. Scale bar: 0.5 cm. **c** The expression levels of candidate genes for AgCC phenotype. Candidate genes located within the Quantitative Trait Loci for callosum phenotype were reported by the previous study^3^. These genes were analyzed by qPCR to compare their levels in BTBR/R and BTBR/J in postnatal day 5 brain. **d** qPCR analysis for Draxin mRNA levels in both BTBR strains. **e** Upper, Sequence alignment of Draxin cDNA cloned from BTBR/R or BTBR/J postnatal day 8 brain to B6. Bottom, An 8-bp deletion in exon 2 was found in draxin from BTBR/J, which caused a premature stop codon. **f** Western blotting for Draxin protein levels in BTBR/R and BTBR/J with biological triplicate. Arrowhead indicates the molecular weight of 58 KD. Graphs show the mean (± or +S.E.M.) for each strain and analyzed by one-way ANOVA with Tukey’s post-hoc test, *, p<0.05; **, p<0.01; ***, p<0.001.

**Fig. S3**

**
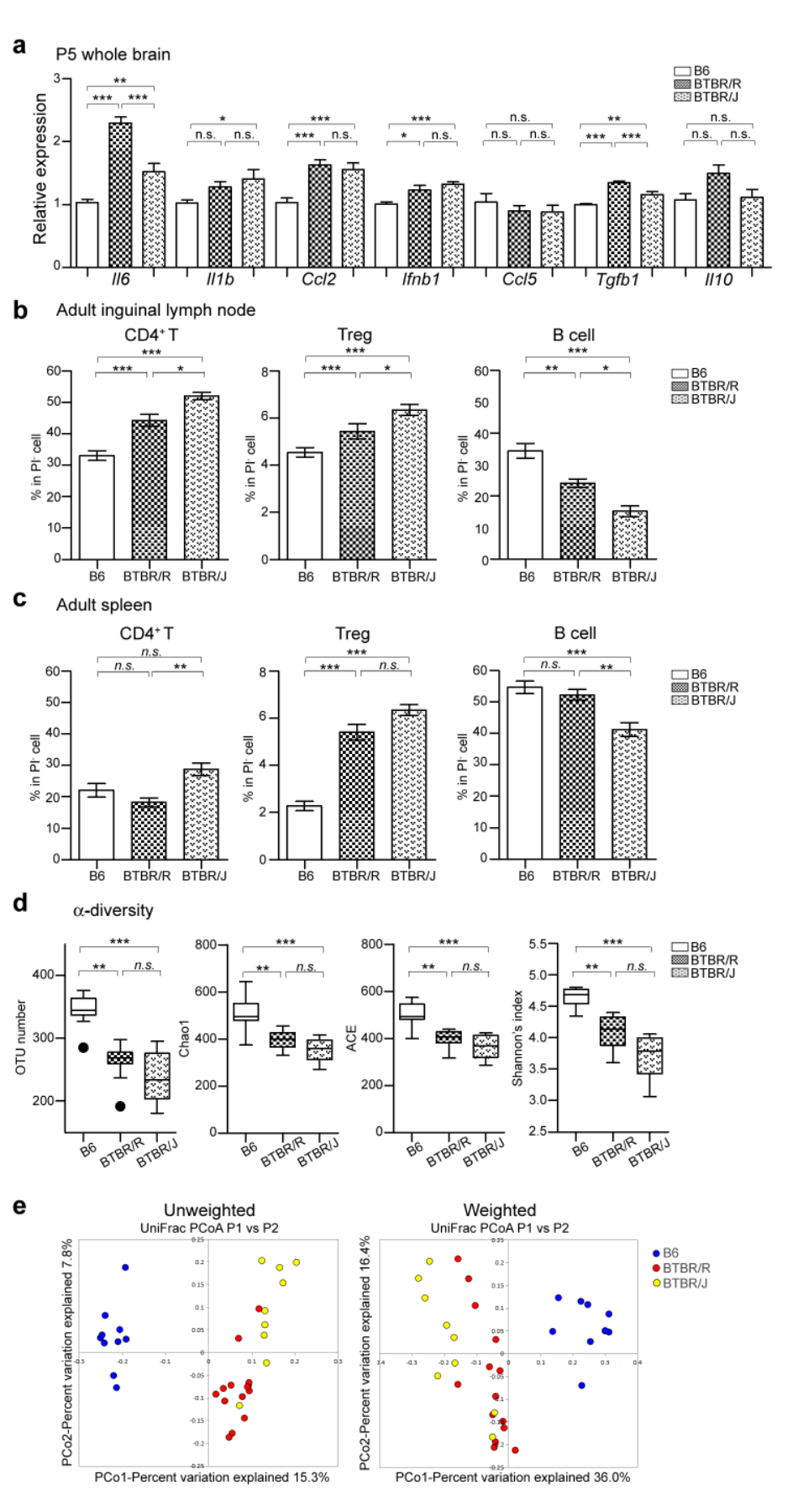
**

**Supplementary Fig. S3 BTBR/R also showed systemic immune dysregulation and the associated comorbid symptom of gut dysbiosis. a** The relative expression levels of inflammatory cytokines in postnatal day 5 brains were analyzed by qPCR in 3 strains. B6, n=14; BTBR/R, n=10; BTBR/J, n=9. One-way ANOVA (effect of genotype), *Il6*, F_2,43_= 50.35, P<0.0001; *Il1b*, F_2,43_= 4.468, P=0.0173; *Ccl2*, F_2,43_= 15.89, P<0.0001; *Ifnb*, F_2,43_= 9.382, P=0.0004; *Ccl5*, F_2,43_= 0.5916, P=0.5577; *Tgfb1*, F_2,43_= 35.05, P<0.0001; *Il10*, F_2,43_= 3.941, P=0.0268 followed by Tukey's multiple comparison test, ***, *p*<0.001; **, *p*<0.01; *, *p*<0.05; n.s, not significant). Data shown are mean (± or + S.E.M.). **b** FACS analysis of T and B cell population in peripheral immune organs of the inguinal lymph node in 3 strains. B6, n=14; BTBR/R, n=10; BTBR/J, n=9. One-way ANOVA (effect of genotype), CD4^+^ T cells, F_2,30_= 37.65, P<0.0001; Treg, F_2,30_= 37.65, P<0.0001; B cells, F_2,30_= 23.20, P<0.0001 followed by Tukey's multiple comparison test, ***, *p*<0.001; **, *p*<0.01; *, *p*<0.05; n.s, not significant). Data shown are mean (± or + S.E.M.). **c** FACS analysis of B and T cell population in peripheral immune organs of the spleen in 3 strains. B6, n=14; BTBR/R, n=12; BTBR/J, n=9. One-way ANOVA (effect of genotype), CD4^+^ T cells, F_2,32_= 6.821, P=0.0033; Treg, F_2,32_= 69.41, P<0.0001; B cells, F_2,32_= 11.43, P=0.0001 followed by Tukey's multiple comparison test, ***, *p*<0.001; **, *p*<0.01; *, *p*<0.05; n.s, not significant). Data shown are mean (± or + S.E.M.). **d** Indices indicated the diversity of microbiota composition in the cecal content of 12-wk-old mice. From left to right: Number of OTUs, α-diversity of Chao1, ACE, and Shannon’s index. Bacteria in the cecum were analyzed for 3000 reads in each sample. Box plots show the interquartile ranges (boxes), maximum/minimum values (whiskers), medians (central horizontal lines) and outliers (black dots). B6, n=10; BTBR/R, n=14; BTBR/J, n=9. Kruskal-Wallis test with Dunn’s post-hoc test, number of OTUs, P<0.0001; Chao1, P<0.0001; ACEs, P<0.0001; Shannon’s index,　P<0.0001; ***, *p*<0.001; **, *p*<0.01; *, *p*<0.05; n.s., not significant). **e** β-diversity of microbiota composition in the cecal content of 12-wk-old mice. Unweighted (left) and weighted (right) UniFrac-based 2D PCoA plot constructed for all OTUs. B6, n=10; BTBR/R, n=14; BTBR/J, n=9. PERMANOVA, unweighted (left), *p* = 0.001; weighted (right), *p* = 0.001).

**Fig. S4**

**
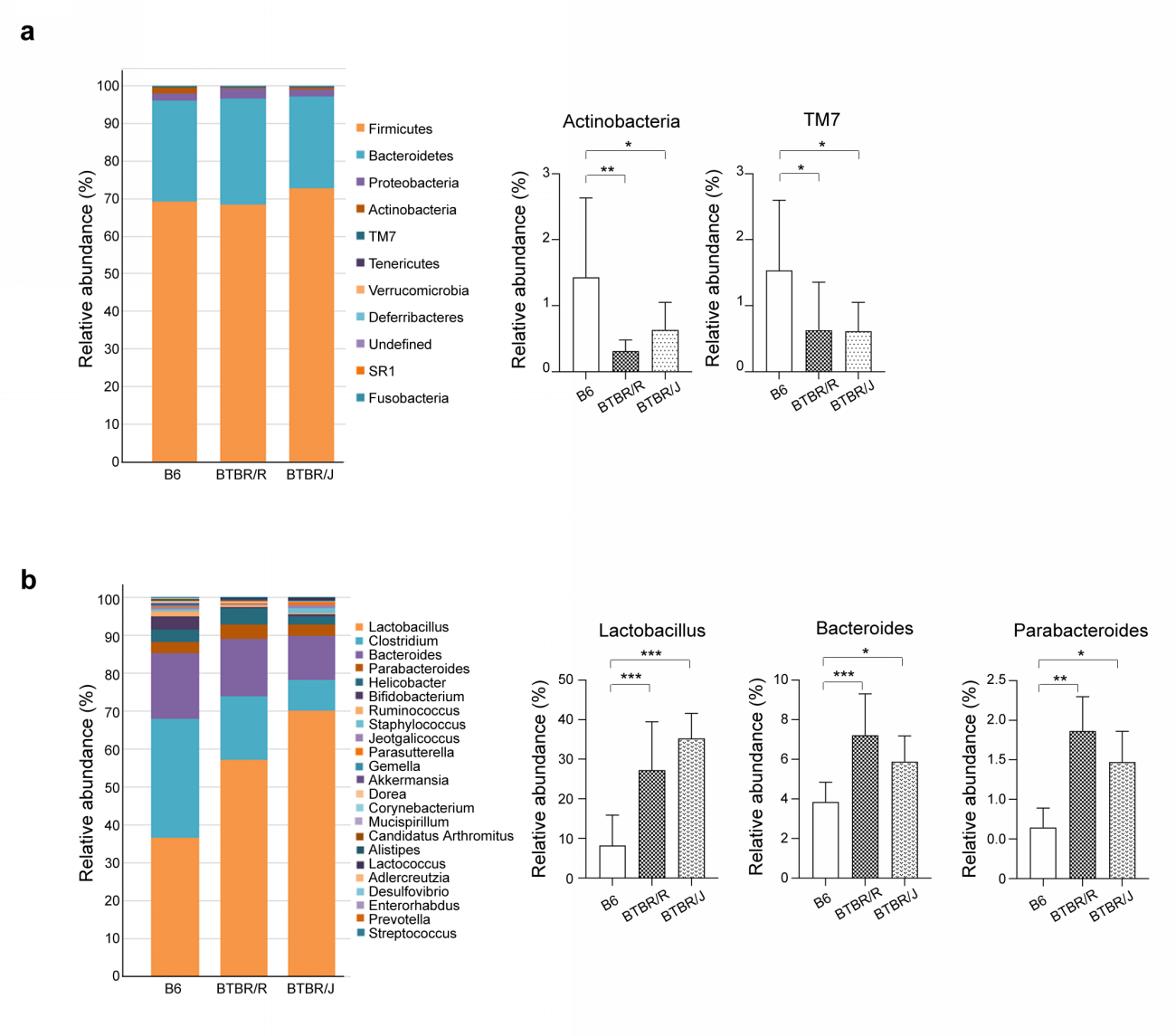
Supplementary Fig. S4 Cecal microbiota composition at Phylum and Genus levels in 12-wk-old male, B6, BTBR/R, and BTBR/J mice. a** Left, the relative abundance of all identified OTUs was classified at the phylum level. Right, microbiota of BTBR/R and BTBR/J mice showed similar differences in the phylum of Actinobacteria and TM7. Graphs show mean + s.e.m (n=10, 15 and 9 for B6, BTBR/R and BTBR/J; One-way ANOVA (effect of strain), Actinobacteria, F_2,30_= 7.501, P=0.0023; TM7, F_2,30_= 4.655, P=0.0173; followed by Tukey's multiple comparison test, ***, *p*<0.001; **, *p*<0.01; *, *p*<0.05).  **b** Left, the relative abundance of all identified OTUs was classified at the genus level. Right, microbiota of BTBR/R and BTBR/J mice showed similar differences in the genus of Lactobacillus, Bacteroides, and

Parabacterioides. Graphs show mean + s.e.m (n=10, 15 and 9 for B6, BTBR/R and BTBR/J). **Fig. S5**

**
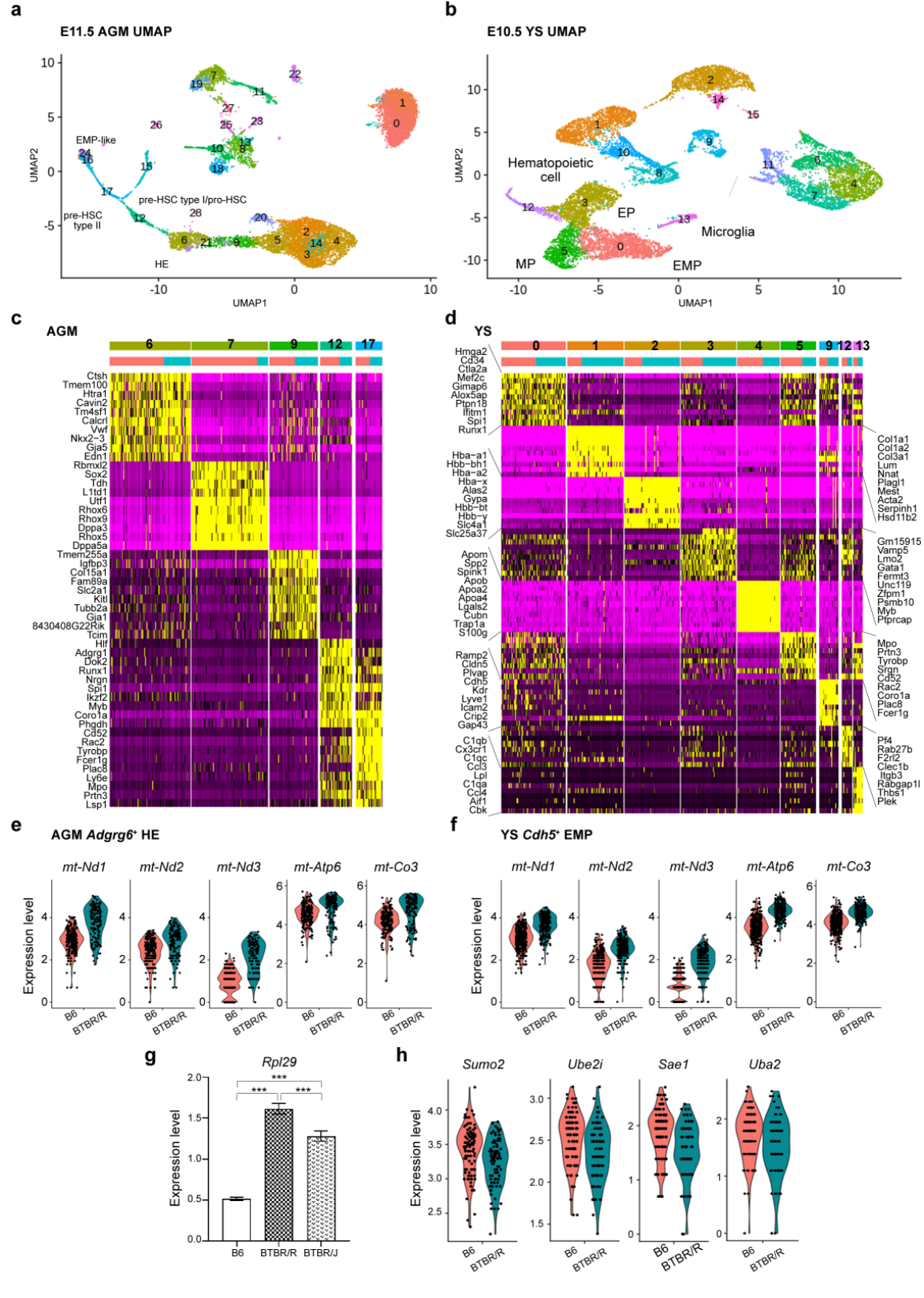
**

**Supplementary Fig. S5 Single-cell RNA-seq analysis of E11.5 AGM and E10.5 YS^1^. a** Unbiased clustering of E11.5 AGM cells from B6 and BTBR/R mice was analyzed by Seurat. **b** Unbiased clustering of E10.5 YS cells from B6 and BTBR/R mice was analyzed by Seurat. **c** Heatmap of top 10 genes enriched in the selected AGM cell clusters for bubble plot (Fig. 3a). Cells under the pink bar, B6; cells under the blue bar, BTBR. **d** Heatmap of top 10 genes enriched in the selected YS cell clusters for bubble plot (Fig. 3b). Cells under the pink bar, B6; cells under the blue bar, BTBR. **e** AGM *Adgrg6*^+^ HE (hemogenic endothelium). The anabolic processes of oxidative phosphorylation for energy production in mitochondria were more activated in BTBR/R mice. (Wilcoxon rank-sum test with Benjamini Hochberg correction, FDR<0.05; all genes are *p*<0.0001). **f** YS *Cdh5*^+^ EMP (erythro-myeloid progenitor). The anabolic processes of oxidative phosphorylation for energy production in mitochondria were more activated in BTBR/R mice. (Wilcoxon rank-sum test with Benjamini Hochberg correction, FDR<0.05; all genes are *p*<0.0001). **g** The expression of ribosomal subunits *Rpl29,* a molecular signature of viral infection, was significantly increased in BTBR/R and BTBR/J testis. One-way ANOVA (effect of genotype), F_2,18_= 111.4, P<0.001. Data shown are mean (±S.E.M.) for each strain and analyzed by one-way ANOVA followed by Tukey's multiple comparison test, ***, *p*<0.001). **h** The expression of sumoylation factors, which silence Class II ERV expression, was reduced in BTBR/R mice (Wilcoxon rank-sum test with Benjamini-Hochberg correction, FDR<0.05; *Sumo2*, *p*=0.0068; *Ube2i*, *p*=0.0076; *Sae1*, *p*=0.0009, except *Uba2*, *p*=0.0726).

**Fig. S6**

**
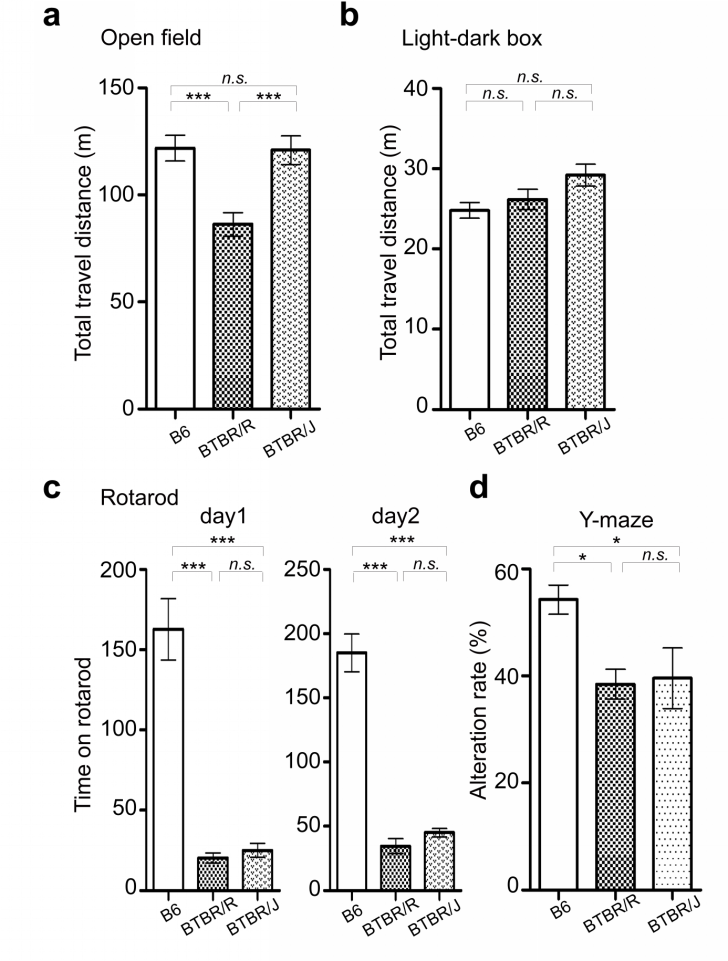
**

**Supplementary Fig. S6 BTBR/R showed behavioral deficits and autistic phenotypes differently than BTBR/J.** All the behavioral tests were analyzed in both BTBR strains and compared to B6. **a** Open field test in a 30-minute section. Total travel distance accessed the locomotor activity of the tested subjects. B6, n=17; BTBR/R, n=22; BTBR/J, n=14. One-way ANOVA (effect of genotype), F_2,50_= 12.62, P<0.0001. **b** Light-dark box in a10-minute section. Total travel distance accessed the locomotor activity of the tested subjects. B6, n=18; BTBR/R, n=17; BTBR/J, n=8. One-way ANOVA (effect of genotype), F_2,40_= 2.564, P=0.0896. **c** Rotarod test for two consecutive days. B6, n=5; BTBR/R, n=10; BTBR/J, n=3. Day 1, one-way ANOVA (effect of genotype), F_2,15_= 65.62, P<0.0001. Day 2, one-way ANOVA (effect of genotype), F_2,15_= 79.77, P<0.0001. **d** Y-maze test for short-term/working memory. B6, n=5; BTBR/R, n=4; BTBR/J, n=4. One-way ANOVA (effect of genotype), F_2,10_= 5.758, P=0.0217. Data shown are mean (±S.E.M.) for each strain and analyzed by one-way ANOVA followed by Tukey's multiple comparison test, ***, *p*<0.001; **, *p*<0.01; *, *p*<0.05; n.s., not significant).

**Supplemental Reference**

1. Lin CW, Septyaningtrias DE, Chao HW, Konda M, Atarashi K, Takeshita K *et al.* A common epigenetic mechanism across different cellular origins underlies systemic immune dysregulation in an idiopathic autism mouse model. *Molecular psychiatry* 2022.

2. Tamada K, Fukumoto K, Toya T, Nakai N, Awasthi JR, Tanaka S *et al.* Genetic dissection identifies Necdin as a driver gene in a mouse model of paternal 15q duplications. *Nature communications* 2021; **12**(1)**:** 4056.

3. Moy SS, Nadler JJ, Young NB, Perez A, Holloway LP, Barbaro RP *et al.* Mouse behavioral tasks relevant to autism: phenotypes of 10 inbred strains. *Behavioural brain research* 2007; **176**(1)**:** 4-20.

4. Jones-Davis DM, Yang M, Rider E, Osbun NC, da Gente GJ, Li J *et al.* Quantitative trait loci for interhemispheric commissure development and social behaviors in the BTBR T(+) tf/J mouse model of autism. *PloS one* 2013; **8**(4)**:** e61829.

5. Islam SM, Shinmyo Y, Okafuji T, Su Y, Naser IB, Ahmed G *et al.* Draxin, a repulsive guidance protein for spinal cord and forebrain commissures. *Science* 2009; **323**(5912)**:** 388-393.

6. Zhang S, Su Y, Shinmyo Y, Islam SM, Naser IB, Ahmed G *et al.* Draxin, a repulsive axon guidance protein, is involved in hippocampal development. *Neuroscience research* 2010; **66**(1)**:** 53-61.

7. Meltzer A, Van de Water J. The Role of the Immune System in Autism Spectrum Disorder. *Neuropsychopharmacology : official publication of the American College of Neuropsychopharmacology* 2017; **42**(1)**:** 284-298.

8. Needham BD, Tang W, Wu WL. Searching for the gut microbial contributing factors to social behavior in rodent models of autism spectrum disorder. *Developmental neurobiology* 2018; **78**(5)**:** 474-499.

9. Heo Y, Zhang Y, Gao D, Miller VM, Lawrence DA. Aberrant immune responses in a mouse with behavioral disorders. *PloS one* 2011; **6**(7)**:** e20912.

10. Onore CE, Careaga M, Babineau BA, Schwartzer JJ, Berman RF, Ashwood P. Inflammatory macrophage phenotype in BTBR T+tf/J mice. *Frontiers in neuroscience* 2013; **7:** 158.

11. Coretti L, Cristiano C, Florio E, Scala G, Lama A, Keller S *et al.* Sex-related alterations of gut microbiota composition in the BTBR mouse model of autism spectrum disorder. *Scientific reports* 2017; **7:** 45356.

12. Golubeva AV, Joyce SA, Moloney G, Burokas A, Sherwin E, Arboleya S *et al.* Microbiota-related Changes in Bile Acid & Tryptophan Metabolism are Associated with Gastrointestinal Dysfunction in a Mouse Model of Autism. *EBioMedicine* 2017; **24:** 166-178.
